# Enhanced Qualities of High-Density Lipoproteins (HDLs) with Antioxidant Abilities Are Associated with Lower Susceptibility of Hypertension in Middle-Aged Korean Participants: Impaired HDL Quality and Hypertension Risk

**DOI:** 10.3390/ijms27021108

**Published:** 2026-01-22

**Authors:** Kyung-Hyun Cho, Chae-Eun Yang, Sang Hyuk Lee, Yunki Lee, Ashutosh Bahuguna

**Affiliations:** Raydel HDL Research Institute, Medical Innovation Complex, Daegu 41061, Republic of Korea

**Keywords:** high-density lipoproteins, HDL, glucose, glycation, oxidation, paraoxonase, PON, Ferric ion reduction ability, FRA, hypertension

## Abstract

The quality of high-density lipoproteins (HDLs) is characterized by lipid and protein composition, oxidation and glycation extent, and particle size, while the quantity of HDL-C is just the cholesterol amount in HDL. The inverse association between HDL-C and cardiovascular disease (CVD) and hypertension has been well established; however, the U-shaped mortality risk observed from HDL-C underscores that HDL quality and function are equally important. The present cross-sectional study assessed the correlations of serum lipid and glucose profiles, and low-density lipoprotein (LDL) and HDL characteristics, with blood pressure (BP) distribution in ordinary middle-aged Korean participants (n = 50; mean age 47.0 ± 11.7 years; males: n = 25, 49.2.0 ± 11.7 years; females: n = 25, 44.8 ± 11.5 years), with particular focus on HDL quality and its antioxidant capacity. This study observed that serum elevated triglyceride (TG) and glucose levels were directly proportional to elevated systolic BP (SBP) and diastolic BP (DBP), whereas serum total cholesterol (TC), LDL-C, and HDL-C were not correlated with BP. However, HDL-C/TC (%) was negatively associated with SBP (*p* = 0.036), while TG/HDL-C and glucose/HDL-C ratios were positively associated with both SBP and DBP, suggesting that TG and glucose proportions relative to HDL-C are probable predictors of hypertension. Elevations of TG, oxidation, and glycation in LDL were positively associated with elevations of BP, whereas LDL particle size was negatively correlated with BP. Similarly, elevations of TG and glycation in HDL_2_ and HDL_3_ were positively correlated with elevations of BP, while the particle size of HDL_2_ was negatively correlated with BP. The heightened HDL_2_-associated paraoxonase (PON) activity and ferric ion reduction ability (FRA) negatively correlated with LDL oxidation and particle size, whereas elevated HDL_3_-associated PON and FRA activities were inversely related to LDL glycation. An enhanced glycation in HDL_2_ was negatively correlated with HDL_2_-associated PON activity and FRA, while an increase in HDL_2_ particle size was only dependent on the associated PON activity but not on FRA. In conclusion, observational outcomes demonstrated that improved HDL quality and functionality (characterized by large particle size, reduced glycation, and higher FRA and PON activities) were inversely correlated with LDL oxidation, glycation, particle shrinkage, and the risk of hypertension.

## 1. Introduction

It is well known that serum high-density lipoprotein cholesterol (HDL-C) levels are inversely associated with the risk of cardiovascular disease, hypertension, stroke, and diabetes mellitus [1,2,3]. Also, higher HDL-C levels are linked to a lower incidence of metabolic syndrome [4]. This inverse relationship between blood pressure (BP) and HDL-C has been observed in many national health and nutrition examination surveys, in which individuals with higher HDL-C have lower systolic BP (SBP) and diastolic BP (DBP) [5,6]. Nevertheless, it has also been reported that extremely high levels of HDL-C are not always beneficial and can be associated with an increased risk of cardiovascular disease (CVD) and mortality [7,8]. A U-shaped relationship between HDL-C and all-cause mortality suggests that extremely low and high HDL-C levels are associated with increased mortality, while moderate to high levels of 73 mg/dL and 93 mg/dL for men and women, respectively, are associated with the lowest all-cause mortality [9]. These studies suggest that HDL quality, rather than HDL-C quantity, may play a more important role in determining its beneficial functions. This notion is supported by evidence showing that excessively high HDL-C levels are associated with increased all-cause mortality [10,11]. Moreover, studies have shown that poor-quality or dysfunctional HDL can exacerbate pro-atherogenic processes, thereby increasing the risk of cardiovascular mortality and hypertension, particularly in men [12,13].

High-quality HDL is characterized by higher cholesterol and apolipoprotein (apo)A-I content, spherical morphology, larger particle size, and resistance to glycation and oxidative damage [14]. Its functionality is mainly reflected in strong antioxidant capacity, such as paraoxonase (PON) activity and ferric ion reduction ability (FRA), and effective cholesterol efflux from atherosclerotic plaque [7]. In contrast, dysfunctional HDL is triglyceride (TG)-enriched, smaller, morphologically distorted, and more susceptible to glycation and oxidation [15], with reduced apoA-I content, weakened FRA and PON activities, and impaired cholesterol efflux capacity [16]. Consequently, dysfunctional HDL fails to protect LDL from oxidation and glycation, and it increases the formation of small dense LDL (sdLDL) [17]. These sdLDL particles are highly atherogenic due to their enhanced susceptibility to oxidative and glycation stress [18], enriched in fatty acids in TG, and depleted in antioxidant activity [19,20], with apolipoprotein (apo) B-100 fragmentation and reduced particle size [21]. Both sdLDL and glycoxidized LDL are independent risk factors for CVD and metabolic disease. Although HDL is known to protect LDL, the specific roles of HDL_2_ and HDL_3_ in preventing LDL from glycation and oxidation insult have not been fully elucidated.

Prior studies have focused primarily on the impact of HDL quality on atherosclerotic outcomes, and not much on the hypertension risk. Furthermore, population-specific evidence is critical in cardiometabolic research, as HDL function and lipoprotein profiles vary across ethnicities and metabolic backgrounds [22,23]. Specifically, studies with Korean participants with HDL functionality and hypertension are not extensively conducted. In this regard, the present study attempts to explore the HDL functionality–sdLDL–hypertension axis in the middle-aged Korean population.

In the current study, we examined the correlations of serum lipids, glucose profile, and lipoprotein (LDL, HDL_2_, and HDL_3_) properties with SBP and DBP in middle-aged Korean participants. Moreover, this study investigates the influence of HDL quality and functionality parameters, including particle size, cholesterol and TG content, glycation extent, and antioxidant abilities, on the incidence of pre-hypertension.

## 2. Results

### 2.1. Distribution of Age, Blood Pressure, and Body Mass Index

All participants showed normal distribution with a middle-aged population around 47.0 ± 11.7 years without differences between genders (Table 1). The combined BP of the participants showed a pre-hypertension stage of SBP around 126 ± 15.8 mmHg; however, the outcomes of the female group showed a normotensive range of SBP and DBP. The male group showed 11% and 14% higher SBP and DBP, respectively, than those of the female group. Anthropometric analysis revealed that the male group showed an obese range (BMI ≥ 25), with a 1.2-fold higher body mass index (BMI) than the female group, which was in the normal range, although the cumulative BMI from the total participants showed an overweight range based on the classification of the Asian-Specific BMI guidelines (>BMI 23.0–24.9). The female group showed 12% higher body fat (%) than the male group (*p* = 0.019); however, the male group showed 61% higher visceral fat than the female group (*p* < 0.001).

### 2.2. Correlation of Blood Lipid Profiles and Blood Pressure

**Figure 1:**
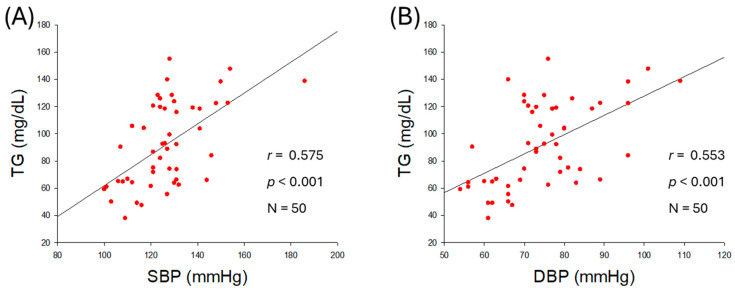

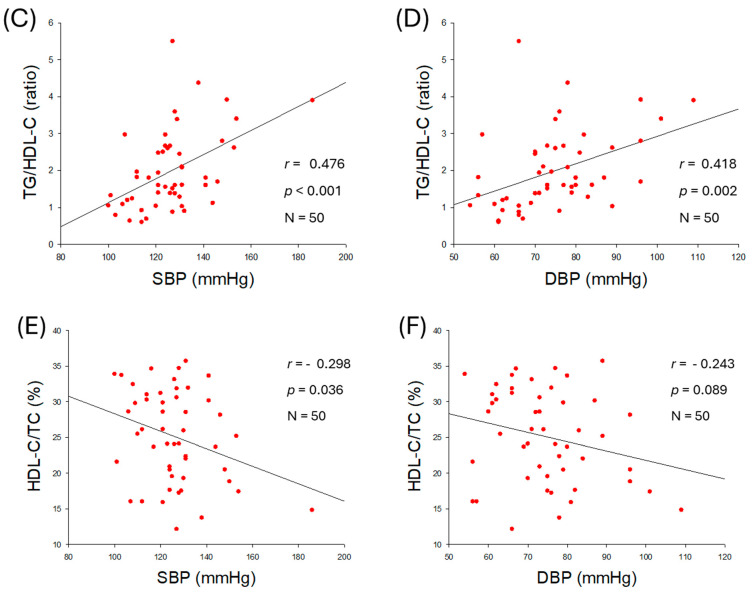
Correlation analysis of serum lipid profile and blood pressure (BP). (**A**) Regression analysis between serum triglyceride (TG) and systolic blood pressure (SBP). (**B**) Regression analysis between serum TG and diastolic blood pressure (DBP). (**C**) Regression analysis between serum TG/high-density lipoprotein cholesterol (HDL-C) and SBP. (**D**) Regression analysis between serum TG/HDL-C and DBP. (**E**) Regression analysis between serum HDL-C/total cholesterol (TC) (%) and SBP. (**F**) Regression analysis between serum HDL-C/TC (%) and DBP.

As shown in Figure 1A,B, an increase in serum TG was positively correlated with an increase in both SBP (*r* = 0.575, *p* < 0.001) and DBP (*r* = 0.553, *p* < 0.001), although an increase in serum TC was not correlated with an increase in SBP (*r* = 0.222, *p* = 0.121) and DBP (*r* = 0.117, *p* = 0.419) (Table 2, Supplementary Figure S1A,B). Similar to TC, a non-significant correlation of LDL-C was noticed with SBP and DBP (Supplementary Figure S1C,D). A negative non-significant correlation between serum HDL-C levels and SBP (*r* = −0.238, *p* = 0.096) and DBP (*r* = −0.234, *p* = 0.102) was observed (Supplementary Figure S1E,F). The serum TG/HDL-C ratio was significantly and positively correlated with SBP (*r* = 0.476, *p* < 0.001) and DBP (*r* = 0.418, *p* = 0.002) as shown in Figure 1C,D. The HDL-C/TC (%) was negatively correlated with only SBP with significance (*r* = −0.298, *p* = 0.036), although HDL-C/TC (%) showed a non-significant correlation (*r* = −0.243, *p* = 0.089) with DBP. These results revealed that BP was more closely associated with serum TG (mg/dL), TG/HDL-C (ratio), and HDL-C/TC (%) rather than the individual levels of serum TC (mg/dL) or HDL-C (mg/dL). The outcomes suggesting a mutual ratio of TC, TG, and HDL-C are more important than the absolute and independent quantity of TC, TG, and HDL-C.

### 2.3. Correlation of Serum Glucose and Blood Pressure

**Figure 2:**
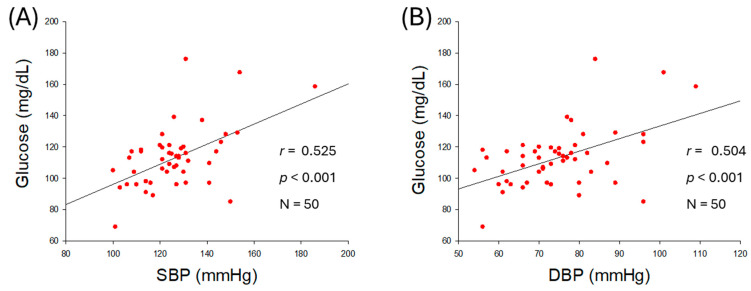

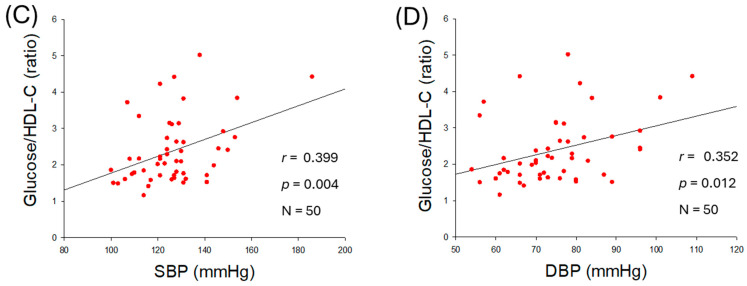
Correlation analysis of serum glucose level and blood pressure (BP). (**A**) Regression analysis between serum glucose level and systolic blood pressure (SBP). (**B**) Regression analysis between serum glucose level and diastolic blood pressure (DBP). (**C**) Regression analysis between serum glucose/high-density lipoprotein cholesterol (HDL-C) ratio and SBP. (**D**) Regression analysis between serum glucose/HDL-C ratio and DBP.

As shown in Figure 2A,B, serum glucose level was positively associated with an increase in SBP (*r* = 0.525, *p* < 0.001) and DBP (*r* = 0.504, *p* < 0.001), suggesting that hyperglycemia is associated with hypertension. The ratio of glucose/HDL-C was also positively correlated with SBP (*r* = 0.399, *p* = 0.004) and DBP (*r* = 0.352, *p* = 0.012) as shown in Figure 2C,D, indicating that both hyperglycemia and reduced HDL-C level with respect to blood glucose are intimately associated with the severity of hypertension.

### 2.4. Correlation of LDL Quality and Blood Pressure

**Figure 3:**
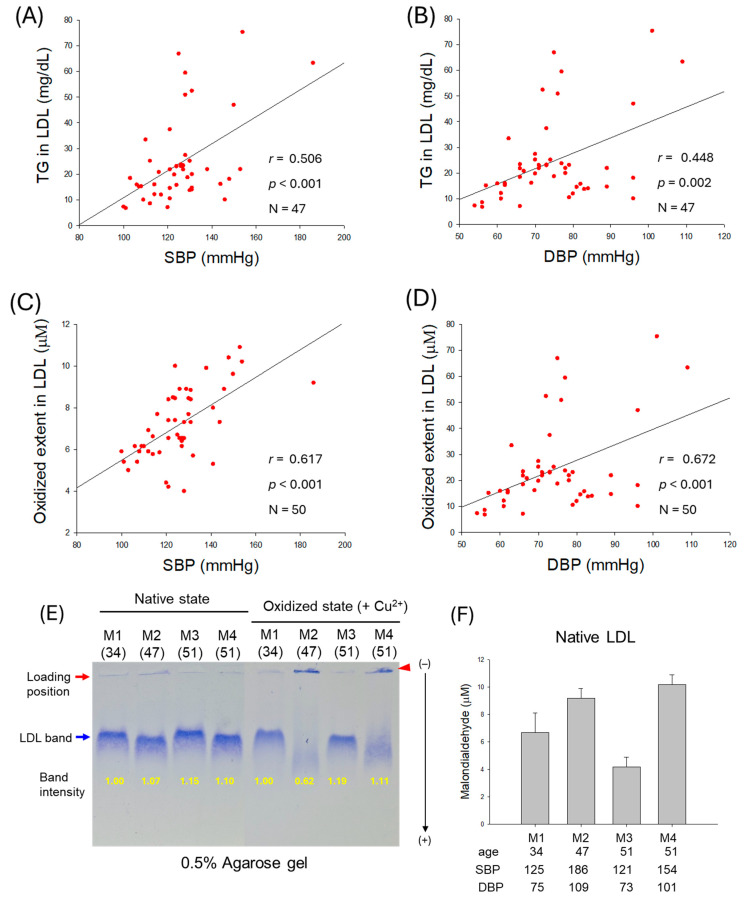
Correlation of low-density lipoprotein (LDL) quality and blood pressure (BP). (**A**,**B**) Regression analysis of triglyceride (TG) content in LDL with systolic blood pressure (SBP) and diastolic blood pressure (DBP), respectively; the data for TG is from only 47 participants, as data from 3 participants showed some inconsistency due to lower volume of LDL. (**C**) Regression analysis of oxidized extent in LDL and SBP. (**D**) Regression analysis of oxidized extent in LDL and DBP. (**E**) Representative electrophoretic patterns of LDL from typical male normotensive participants (M1 and M3, 34- and 51-year-old, respectively) and hypertensive participants (M2 and M4, 47- and 51-year-old, respectively) under native state and oxidized state (in the presence of Cu^2+^). (**F**) Quantification of oxidized species in native LDL using thiobarbituric acid reactive substance (TBARS) assay with malondialdehyde (MDA) standard.

As shown in Figure 3A,B, an increase in TG content in LDL was positively associated with SBP (*r* = 0.506, *p* < 0.001) and DBP (*r* = 0.448, *p* = 0.002), suggesting that an increase in TG in LDL, which reflects impairment of LDL quality, was correlated with hypertension. Moreover, as shown in Figure 3C,D, the oxidized extent of LDL was more positively associated with an increase in SBP (*r* = 0.617, *p* < 0.001) and DBP (*r* = 0.672, *p* < 0.001). These results suggest that high prevalence of oxidized LDL and TG-enriched LDL is associated with exacerbation of hypertension.

As depicted in Figure 3E, electrophoretic analysis of LDL from typical male normotensive participants (M1 and M3) and hypertensive participants (M2 and M4) was performed. The results revealed that hypertensive participants showed weaker band intensity and faster electromobility than normotensive participants in both native and oxidized states (induced by CuSO_4_). Especially in the oxidized state, under CuSO_4_ treatment (final 10 μM), LDL from hypertensive participants almost disappeared, as indicated by the blue arrow, due to the oxidation, while aggregated bands were detected in loading position, as indicated by the red arrowhead. Furthermore, the quantification of oxidized species in LDL using a malondialdehyde (MDA) standard by thiobarbituric acid reactive substances (TBARSs) revealed that hypertensive partcipants showed substantially higher MDA levels than normotensive participants (Figure 3F).

### 2.5. Particle Size and Glycation Extent of LDL

**Figure 4:**
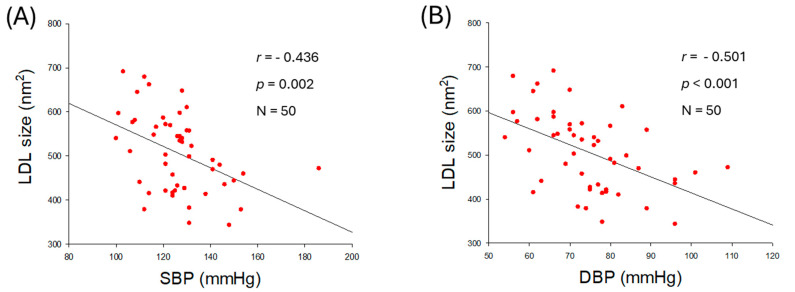

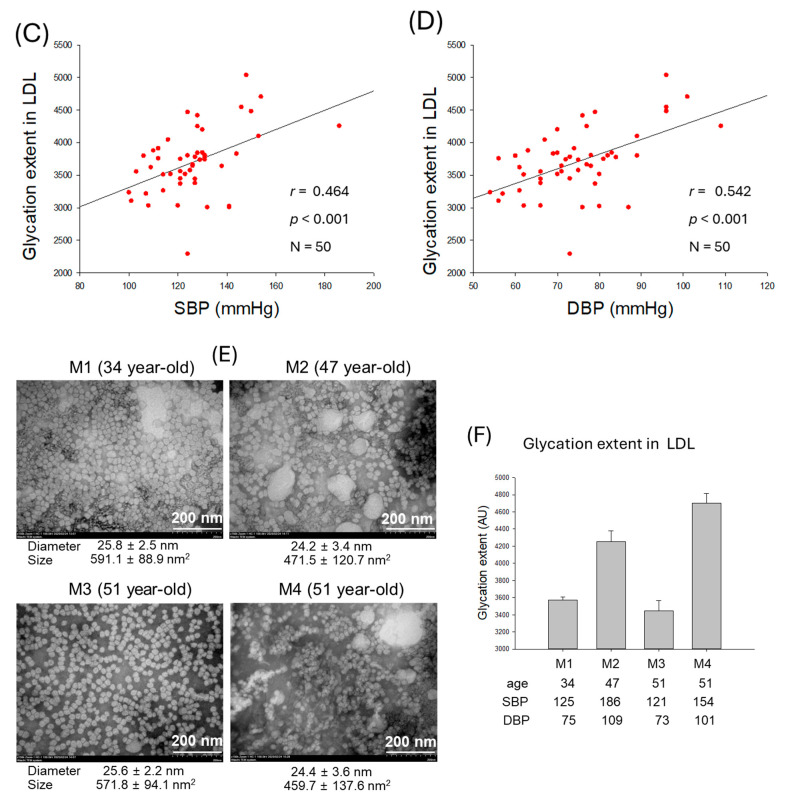
Correlation analysis of qualities of low-density lipoprotein (LDL) particles and blood pressure (BP). (**A**) Regression analysis of LDL particle size and systolic blood pressure (SBP). (**B**) Regression analysis of LDL particle size and diastolic blood pressure (DBP). (**C**) Regression analysis of glycation extent of LDL and SBP. (**D**) Regression analysis of glycation extent of LDL and DBP. (**E**) Transmission electron microscope (TEM) image of representative male participants. (**F**) Glycation extent of LDL from selected male participants. AU, arbitrary unit.

As shown in Figure 4A,B, particle size of LDL was negatively correlated with SBP (*r* = −0.436, *p* = 0.002) and DBP (*r* = −0.501, *p* < 0.001), suggesting that smaller LDL particle size is more associated with risk of hypertension. Furthermore, as shown in Figure 4C,D, glycation extent in LDL was positively associated with an increase in SBP (*r* = 0.464, *p* < 0.001) and DBP (*r* = 0.542, *p* < 0.001), suggesting that higher glycation in LDL is associated with risk of hypertension, especially DBP. These results indicate that a smaller particle size of LDL with higher glycation and oxidation is correlated with an increase in hypertension risk.

The observation of LDL particles using transmission electron microscopy (TEM) revealed that normotensive participants (M1 and M3) showed 1.3-fold larger LDL particles, around 571~591 nm^2^ particle size, than hypertensive participants (M2 and M4), around 459~471 nm^2^ (Figure 4E, Supplementary Figure S2A–D). Furthermore, LDL images from the hypertensive participants (M2 and M4) exhibited smaller particle numbers, aggregation, and ambiguous morphology with distorted particle shape, whereas LDL images from the normotensive participants (M1 and M3) displayed larger particle numbers that are well separated, with clear spherical morphology. The extent of glycation revealed that normotensive participants (M1 and M3) harbor significantly lower glycation than hypertensive participants (M2 and M4) (Figure 4F). These results suggest the poor quality of LDL, marked by smaller particle size, distorted morphology, a higher degree of oxidation, and glycation directly linked to hypertension.

### 2.6. HDL_2_ Quality and Correlation with Blood Pressure

**Figure 5:**
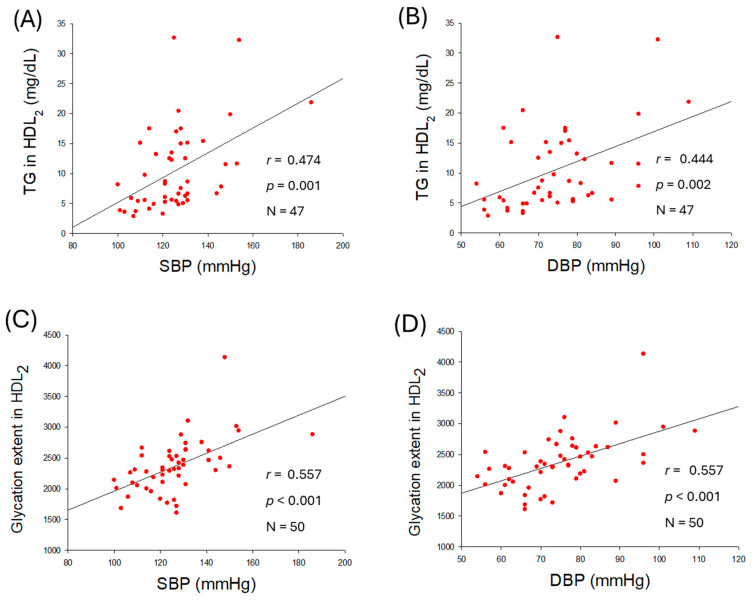
Correlation analysis of high-density lipoprotein (HDL_2_) quality and blood pressure (BP). (**A**,**B**) Regression analysis of triglyceride (TG) content in HDL_2_ with systolic blood pressure (SBP) and diastolic blood pressure (DBP), respectively; the data for TG is from only 47 participants, as data from 3 participants showed some inconsistency due to lower volume of HDL_2_. (**C**) Regression analysis of glycation extent in HDL_2_ and SBP. (**D**) Regression analysis of glycation extent in HDL_2_ and DBP.

As shown in Figure 5A,B, an increase in TG content in HDL_2_ was positively associated with the elevation of SBP (*r* = 0.474, *p* < 0.001) and DBP (*r* = 0.444, *p* = 0.002), while TC content in HDL_2_ was not significantly associated with the elevation of SBP and DBP, although they showed notable negative regression (Supplementary Figure S3A,B). Increased glycation in HDL_2_ was positively associated with the elevation of SBP (*r* = 0.557, *p* < 0.001) and DBP (*r* = 0.557, *p* < 0.001) with remarkable significance (Figure 5C,D), suggesting that higher glycation in HDL_2_ is a risk factor of hypertension.

### 2.7. Correlation of HDL_2_ Size and Blood Pressure

**Figure 6:**
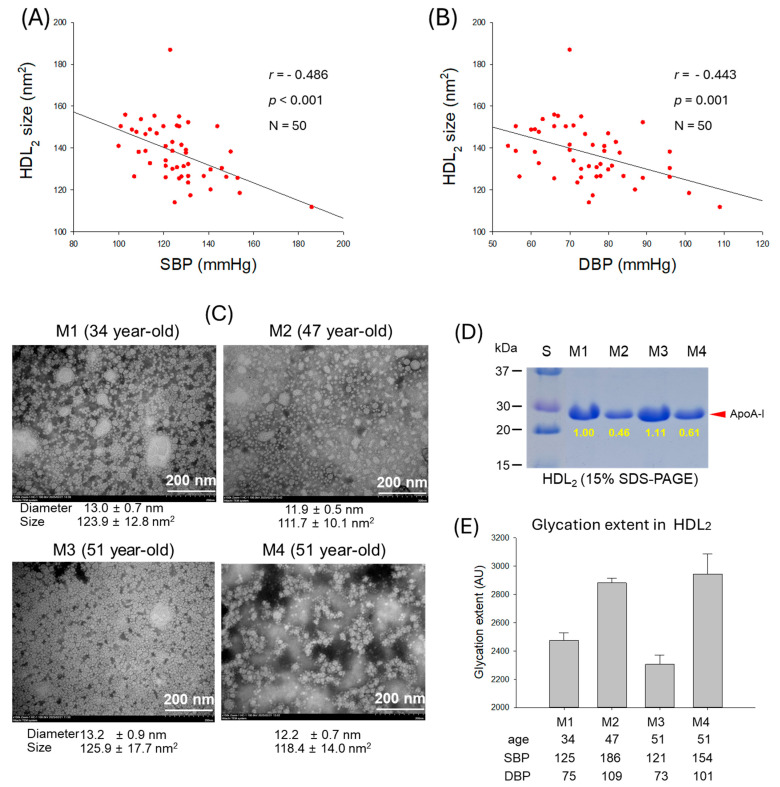
Correlation analysis of high-density lipoprotein (HDL_2_) particle properties and blood pressure (BP). (**A**) Regression analysis of HDL_2_ particle size and systolic blood pressure (SBP). (**B**) Regression analysis of HDL_2_ particle size and diastolic blood pressure (DBP). (**C**) Transmission electron microscope (TEM) images of the representative male participants, comparing between normotensive (M1 and M3) and hypertensive (M2 and M4). (**D**) Electrophoretic profile of HDL_2_ to compare band intensity of apolipoprotein (apo)A-I (15% SDS-PAGE). (**E**) Glycation extent of HDL_2_ from the representative male participants. AU, arbitrary unit.

As shown in Figure 6A,B, particle size of HDL_2_ was negatively correlated with SBP (*r* = −0.486, *p* < 0.001) and DBP (*r* = 0.−443, *p* = 0.001), suggesting that smaller particle size of HDL_2_ is more associated with risk of hypertension. The TEM image revealed that normotensive participants (M1 and M3) showed a bigger size, around 123–126 nm^2^, and distinct particle morphology with higher particle numbers. In contrast, hypertensive participants (M2 and M4) showed a smaller size, around 111–118 nm^2^, with aggregated and ambiguous particle morphology (Figure 6C, Supplementary Figure S4A–D). These results indicate that particle size and morphology of HDL_2_ are correlated with the incidence of hypertension: bigger particle size, more particle numbers, and a clean shape are desirable morphologies for healthy SBP and DBP. Furthermore, electrophoresis (15% SDS-PAGE) of HDL_2_ revealed a remarkably decreased apoA-I band in hypertensive participants (M2 and M4) that was quantified to be around 39~54% less than the apoA-I intensity quantified in the normotensive participants (M1 and M3) (Figure 6D).

**Figure 7:**
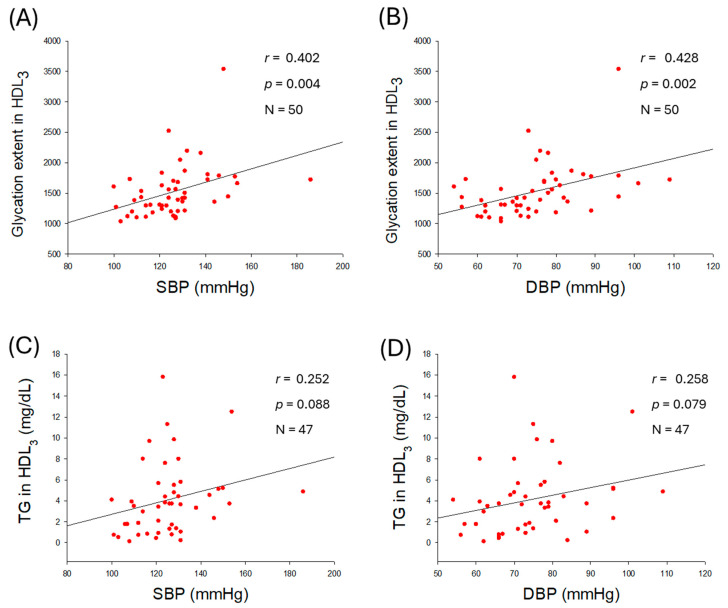
Correlation analysis of high-density lipoprotein (HDL_3_) quality and blood pressure (BP). (**A**) Regression analysis of glycation extent in HDL_3_ with systolic blood pressure (SBP). (**B**) Regression analysis of glycation extent in HDL_3_ and diastolic blood pressure (DBP). (**C**,**D**) Regression analysis of triglyceride (TG) content in HDL_3_ with SBP and DBP, respectively; the data for TG is from only 47 participants, as data from 3 participants showed some inconsistency due to lower volume of HDL_3_.

A lower degree of HDL_2_ glycation was observed in the normotensive participants (M1 and M3), whereas the hypertensive participants (M2 and M4) showed markedly higher glycation levels (Figure 6E). These findings suggest that HDL_2_ glycation is correlated with both SBP and DBP. As depicted in Figure 6E, M2 exhibited a 1.2-fold increase in HDL_2_ glycation compared with M1, which corresponds to the 1.5-fold higher SBP and DBP in these participants. Similarly, M4 displayed a 1.2-fold higher glycation level than M1 and had substantially elevated SBP and DBP values compared with M1. These results support the notion that more glycation in HDL was positively correlated with SBP and DBP as shown in Figure 5C,D and Figure 7A,D.

### 2.8. Glycation Extent in HDL_3_ and Correlation with Blood Pressure

As shown in Figure 7, glycation extent in HDL_3_ was positively correlated with elevation of SBP (*r* = 0.402, *p* = 0.004) and DBP (*r* = 0.428, *p* = 0.002), while TG content in HDL_3_ was associated with non-significant (*p* > 0.05) elevation of BP. These results suggest that an increase in glycation extent in HDL_3_ is a reliable risk marker for hypertension.

### 2.9. Correlation of LDL Qualities and Antioxidant Abilities in HDL_2_

**Figure 8:**
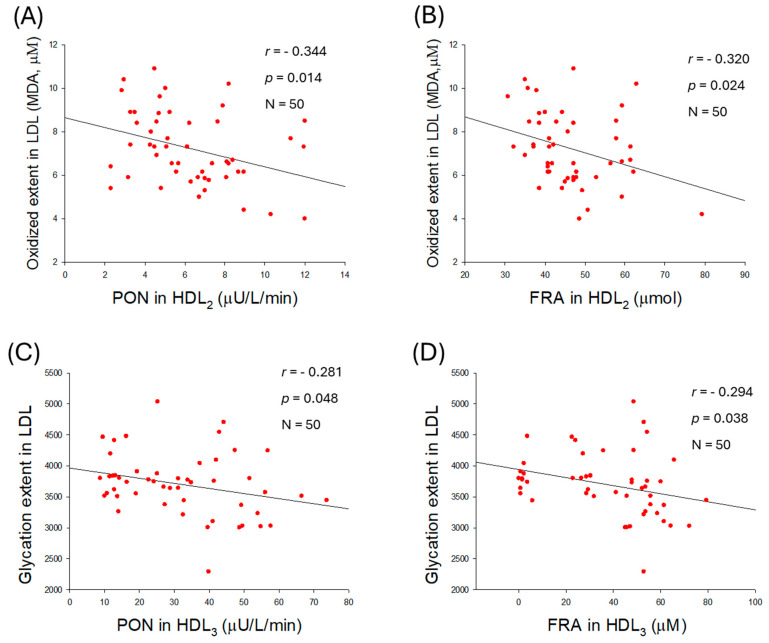

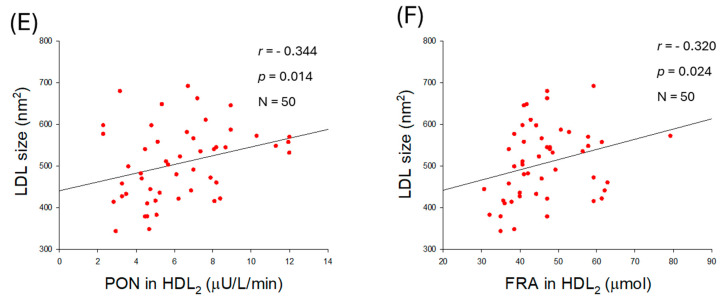
Correlation analysis of low-density lipoprotein (LDL) quality and high-density lipoprotein (HDL)-associated antioxidant activities. (**A**) Regression analysis between oxidized extent in LDL and paraoxonase (PON) in HDL_2_. (**B**) Regression analysis between oxidized extent in LDL and ferric ion reduction (FRA) in HDL_2_. (**C**) Regression analysis between glycation extent in LDL and PON in HDL_3_. (**D**) Regression analysis between glycation extent in LDL and FRA in HDL_3_. (**E**) Regression analysis between LDL size and PON in HDL_2_. (**F**) Regression analysis between LDL size and FRA in HDL_2_.

As shown in Figure 8A,B, the extent of LDL oxidation was negatively correlated with paraoxonase (PON) activity (*r* = −0.344, *p* = 0.014) and ferric ion reduction ability (FRA) in HDL_2_ (*r* = −0.320, *p* = 0.024), whereas HDL_3_-associated PON and FRA were not significantly correlated with the extent of LDL oxidation (Supplementary Figure S5A,B). Interestingly, no correlation was observed between the glycation extent of LDL and HDL_2_-associated PON (*r* = −0.015, *p* = 0.920) or FRA (*r* = −0.131, *p* = 0.364; Supplementary Figure S5C,D). However, a negative correlation was observed between the glycation extent of LDL and PON (*r* = −0.281, *p* = 0.048) activity associated with HDL_3_ (Figure 8C). However, the glycation extent in LDL was non-significantly associated with the FRA (*r* = −0.294, *p* = 0.038) activity of HDL_3_ (Figure 8D).

Furthermore, an increase in LDL size was associated with an enhancement of HDL_2_-associated PON (*r* = −0.344, *p* = 0.014) and FRA (*r* = −0.320., *p* = 0.024) activities (Figure 8E,F). Nevertheless, no correlation of LDL size change was noticed on the HDL_3_-associated PON (*r* = 0.076, *p* = 0.599) and FRA (*r* = 0.004, *p* = 0.976) (Supplementary Figure S5E,F). Collectively, the results indicated that alterations in LDL, such as glycation, oxidation, and smaller particle size, are associated with the antioxidant activity of either HDL_2_ or HDL_3_.

### 2.10. Quality of HDL_2_ and Antioxidant Abilities of HDL_2_

**Figure 9:**
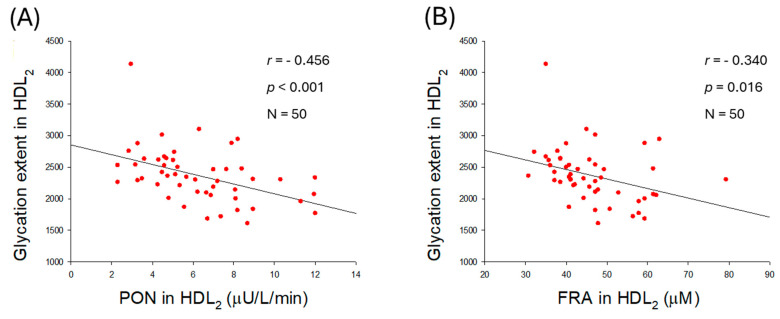

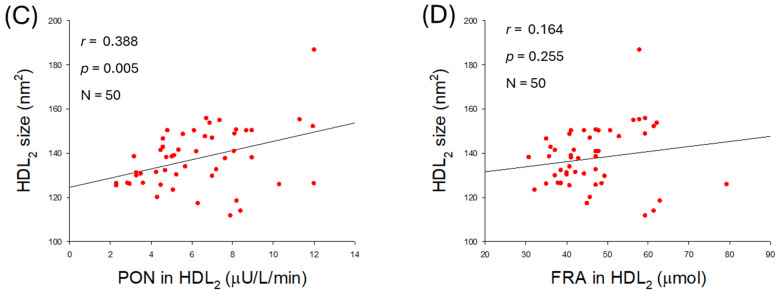
Correlation analysis between the quality of high-density lipoprotein (HDL_2_) and antioxidant abilities of HDL_2_. (**A**) Regression analysis between glycation extent in HDL_2_ and paraoxonase (PON) in HDL_2_. (**B**) Regression analysis between glycation extent in HDL_2_ and ferric ion reduction (FRA) in HDL_2_. (**C**) Regression analysis between particle size of HDL_2_ and PON in HDL_2_. (**D**) Regression analysis between particle size of HDL_2_ and FRA in HDL_2_.

The extent of glycation in HDL_2_ was negatively correlated with the enhancement of PON (*r* = −0.456, *p <* 0.001) and FRA (*r* = −0.340, *p* = 0.016) activities (Figure 9A,B). In addition, the particle size of HDL_2_ was positively associated only with PON activity (*r* = 0.388, *p* = 0.005) but was unrelated to FRA activity (*r* = 0.164, *p* = 0.255). The results indicated that higher PON activity is an important factor associated with reducing glycation and increasing HDL_2_ particle size.

### 2.11. Correlation Analysis of Parameters Between SBP and DBP

As summarized in Table 2, BMI and visceral fat exhibited significant positive correlations with both SBP and DBP. In addition, serum TG, TG/HDL-C, glucose, and glucose/HDL-C levels were also significantly and positively correlated with both SBP and DBP. These results indicate that general obesity and abdominal obesity are important risk factors of hypertension, as elevated TG and glucose levels, along with reduced HDL-C, may contribute to the accumulation of visceral fat in the abdomen.

In LDL, TG content, oxidation extent, and glycated extent were positively correlated with both SBP and DBP, while particle size and diameter were negatively correlated with both SBP and DBP. These results suggest that more oxidized, glycated, TG-enriched, and smaller LDL is directly linked with the incidence of hypertension. In HDL_2_, TG content and glycation extent were positively correlated with both SBP and DBP, while particle size and diameter were negatively correlated with both SBP and DBP. In HDL_3_, only the extent of glycation was positively correlated with elevated SBP and DBP. These results suggest that higher TG content, greater glycation extent, and smaller HDL particle size are correlated with impairment of HDL functionality.

## 3. Discussion

The observational outcomes from the 50 participants, as summarized in Table 2, revealed that several anthropometric and lipid parameters were positively associated with higher BP. Specifically, BMI, visceral fat, serum TG, TG/HDL-C ratio, and serum glucose showed positive associations with increases in BP (Figure 1 and Figure 2). In contrast, serum HDL-C/TC (%) was negatively correlated only with SBP (*r* = −0298, *p* = 0.036), but not with DBP. In addition, weak correlations were observed between age, body fat percentage, subcutaneous fat (kg), and serum LDL-C, and HDL-C with SBP and DBP. These results are in good agreement with a previous report documenting a substantial effect, with the HDL-C/TC (%) ratio associated with a change in SBP, while SBP is well correlated with a decrease in HDL-C [24]. Specifically, women showed a sharper decrease in HDL-C, correlating with increasing SBP, after menopause, according to the Korean National Health and Nutrition Examination Survey in 2017 [24]. A decrease in HDL-C in the female group after middle age was strongly associated with a considerable increase in dementia in later life [25]. The study outcomes are in accordance with previous reports deciphering a deep association of elevated BMI [26] and visceral fat [27] with hypertension. Consistent with present findings, earlier reports showed the impact of dyslipidemia with hypertension [28]. In line with the current findings, a higher TG/HDL-C ratio emerged as a predictor of hypertension [29]. Although earlier reports demonstrated a positive association between TC levels and BP [28], our findings did not show a significant individual effect of TC or HDL-C on BP. Instead, HDL-C in relation to TC (i.e., HDL-C/TC ratio) was inversely associated with BP, highlighting its relevance as a possible predictor of hypertension risk.

In LDL, the increase in TG content was more strongly correlated with the elevation of SBP, with a higher regression coefficient (*r* = 0.506) than with DBP (*r* = 0.448) and higher significance (Figure 3A,B). The increase in oxidized LDL (Figure 3C,D) was more strongly correlated with DBP elevation, with a higher regression coefficient (*r* = 0.672) than with SBP (*r* = 0.617), suggesting that compositional changes in LDL, particularly with respect to TG and oxidation extent, are differently associated with SBP and DBP. Furthermore, as shown in Figure 4 and Table 2, the increase in glycation extent was more strongly associated with an elevation in DBP (*r* = 0.542) than with an elevation in SBP (*r* = 0.464), with greater significance. The decrease in LDL particle size was also associated with an elevation of DBP, with a higher regression coefficient (*r* = −0.501, *p* < 0.001) than with an elevation of SBP (*r* = −0.436, *p* = 0.002) and higher significance. Although the mechanism remains unclear, these results suggest that changes in lipid composition, oxidation extent, glycation extent, and LDL particle size were closely associated with SBP or DBP. Most of the reports depict glycated and oxidized LDL as an important marker of diabetes [30] and coronary heart disease [31,32], though its impact on hypertension is not highlighted much. However, the current observational study gives a preliminary indication of the relationship between elevated BP and glycated and oxidized LDL, highlighting its relevance beyond coronary heart disease.

**Figure 10:**
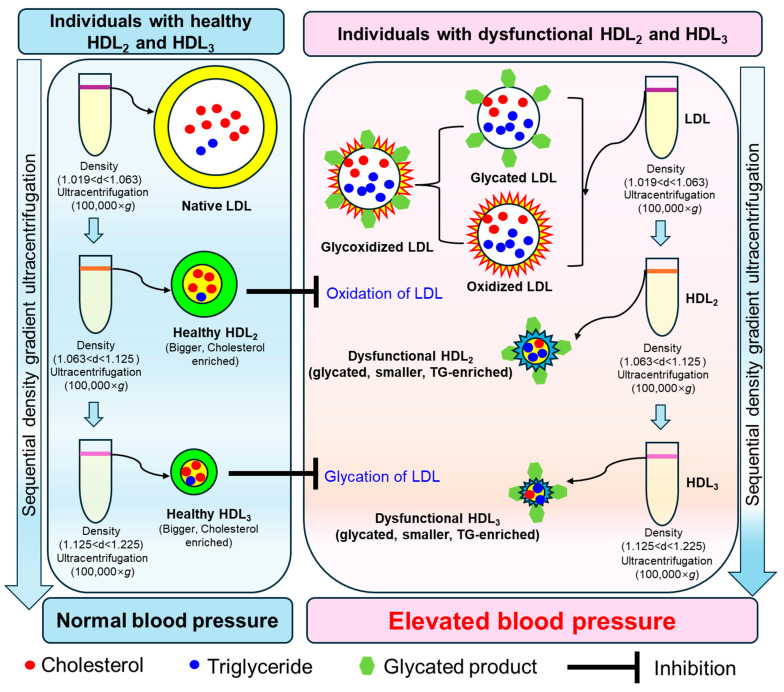
This schematic illustration shows the association between low-density lipoprotein (LDL) and high-density lipoprotein (HDL) subpopulations (HDL_2_ and HDL_3_), including their quality, particle size, and extent of oxidation and glycation, with blood pressure, based on outcomes from a cross-sectional study.

Antioxidant activities of HDL were also negatively correlated with glycated extent; higher glycation content is associated with diminished PON and FRA activities in both HDL_2_ and HDL_3_ (Figure 9A,B). An increase in HDL_2_ particle size is significantly correlated with enhancement of only PON activity (*r* = 0.388, *p* = 0.005) in HDL_2_, not associated with FRA in HDL_2_ (*r* = 0.164, *p* = 0.255). Although LDL particle size was intimately associated with both PON and FRA activities in HDL_2_, HDL_2_ particle size was positively correlated with only higher PON activity, not FRA, in HDL_2_ itself. Collectively, larger HDL particles (both HDL_2_ and HDL_3_) exhibit stronger antioxidant activity, and correlate with protecting LDL (Figure 10). These findings align with the existing literature, which indicates that HDL quality holds greater significance than quantity in CVD risk assessment [33].

On the other hand, higher TG content and greater HDL glycation were positively associated with elevated SBP and DBP, whereas HDL_2_ particle size was negatively correlated with SBP and DBP. These results suggest that TG-enriched, highly glycated, smaller HDL particles are associated with loss of apoA-I and an increased risk of CVD, rather than HDL cholesterol quantity [34,35,36,37]. More interestingly, PON and FRA activities in HDL_2_ were negatively correlated with oxidation extent in LDL, while an increase in LDL particle size was positively correlated with PON and FRA activity in HDL_2_ [38]. Furthermore, the extent of glycation in LDL was negatively correlated with PON and FRA activity in HDL_3_, suggesting distinct roles for HDL_2_ and HDL_3_ associated with LDL oxidation and glycation, respectively. Although a report showed that HDL_3_ exerts a more powerful antioxidative effect than HDL_2_ [39], another report showed that both HDL_2_ and HDL_3_ play an important role in the copper-catalyzed oxidation of LDL [40].

M3 and M4 have the same profession, as professional accountants, and the same age (51 years old), but they showed substantially different blood pressure. A comparison of HDL and LDL quality between the two peers revealed that LDL quality is correlated with HDL quality and functionality, as illustrated in Figure 4. LDL from M3 showed a round shape with distinct and separate distribution and larger particle size around 25.6 ± 2.2 nm. However, LDL from M4 showed a distorted shape, aggregation, and uneven distribution with a smaller particle size of around 24.4 ± 3.6 nm. These results suggest that the morphology of LDL particles, including shape, size, and number, differed between the two men, regardless of the quantity of LDL-C. Strikingly, HDL particles from M3 exhibited a well-defined spherical morphology, uniform dispersion, and a larger average particle size of 13.2 ± 0.9 nm with a higher particle count, as illustrated in Figure 6. In contrast, HDL particles from M4 displayed irregular shapes, aggregation tendencies, less particle numbers, and uneven distribution with smaller average particle size, around 12.2 ± 0.7 nm, with 1.3-fold higher glycation extent.

Although the precise role of HDL associated with glycation is not fully known, research shows that a key protective enzyme in HDL (i.e., PON) loses its activity when exposed to methylglyoxal, creating dysfunctional HDL that can no longer protect against oxidation and inflammation [41]. It is well established that both HDL_2_ and HDL_3_ are correlated with protecting LDL from damage; however, HDL_2_ is more specifically associated with protection from oxidation, while HDL_3_ is more correlated with minimizing glycation, as summarized in Figure 10.

We analyzed, in the current study, various aspects of HDL quality and functionality regarding its correlation with LDL and hypertension in middle-aged participants, especially pre-hypertension in male participants. Major findings of this study are that HDL quality and antioxidant ability are more correlated with the incidence of pre-hypertension than the simple quantity of HDL-C. A larger particle size, enriched cholesterol content, lower TG content, reduced glycation extent, and enhanced antioxidant activity are crucial for vascular health to protect against the incidence of hypertension.

This study includes multiple correlation analyses between the variables and is interpreted as explanatory and hypothesis-generating. The findings provided preliminary information that may be useful for future studies with a larger participant pool with prespecified models.

Limitations and prospects of this study: A small number of participants (n = 50) emerged as a basic limitation of this study to establish a confirmed relationship between HDL functionality, size, and LDL glycation and a risk of hypertension in the Korean population. In addition, the lack of detailed mechanistic insight limits the study’s outcomes. Future studies will focus on large participant pools and on decoding how lipoprotein glycation and antioxidant activities mechanistically lead to hypertension.

## 4. Materials and Methods

### 4.1. Participants

A total of 50 middle-aged volunteers (25 males aged 49.2 ± 11.7 years and 25 females aged 44.8 ± 11.5 years) were randomly recruited via a nationwide advertisement in Korea (2022 to 2023). The study aimed to assess HDL and LDL qualities and quantities. The protocol for human blood donation was conducted according to the latest version of Declaration of Helsinki [42]. The study received ethical approval from the Korea National Institute for Bioethics Policy (KoNIBP, approval number P01-202109-31-009, date of approval 27 September 2021) under the Ministry of Health Care and Welfare (MOHW).

The exclusion criteria were as follows: (1) Maintenance treatment for metabolic disorder, including severe dyslipidemia, hypertension, and diabetes. The exclusion criteria are specifically related to previously diagnosed participants who are receiving ongoing hypertensive treatment. However, newly identified, untreated hypertensive participants at the time of enrolment were included in the study. (2) Severe hepatic, renal, cardiac, respiratory, endocrinological, and metabolic disorder disease. (3) Allergies and autoimmune diseases. (4) Heavy drinkers, more than 30 g of alcohol per day. (5) Taking medicines that may affect lipid metabolism, including raising HDL-C or lowering LDL-C concentration, and lowering triglyceride concentration. (6) Current smoker. (7) Women in pregnancy, in lactation, or planning to become pregnant during the study period. (8) Persons who donated more than 200 mL of blood within one month or 400 mL of blood within three months before starting this study. (9) Others considered unsuitable for the study at the discretion of the principal investigator.

### 4.2. Anthropometric Analysis

The blood pressure (BP) was measured using an Omron HBP-9020 (Kyoto, Japan). Based on the measured BP, participants were categorized into three groups, namely normotensive (<120 mmHg DBP and <80 mmHg SBP), pre-hypertensive (120–139 mmHg DBP and 80–89 mmHg SBP), and hypertensive (>140 mmHg DBP and >90 mmHg SBP) [43]. The height, body weight, body mass index (BMI), body water, total body fat (%), total body fat mass (kg), and visceral fat mass (VFM) (kg) were measured individually using an X-scan plus II body composition analyzer (Jawon Medical, Gyeongsan, Republic of Korea).

### 4.3. Blood Analysis

After an overnight fast, blood was collected using a Vacutainer (BD BioSciences, Franklin Lakes, NJ, USA) without the addition of an anti-coagulant. The serum parameters, as shown in Table 1, were determined by an automatic analyzer (CobasC502; Roche, Germany) at a commercial diagnostic service via SCL Healthcare (Seoul, Republic of Korea). For all the blood analysis parameters, researchers were blinded to the allocated group. Samples were coded with a non-revealing numerical code prior to the analysis.

### 4.4. Isolation of Lipoproteins

Sequential density gradient ultracentrifugation was performed to isolate serum lipoprotein fractions LDL, HDL_2_, and HDL_3_ [44]. In brief, 5 mL of serum from each individual was separately mixed with a density gradient solution (d < 1.019 g/mL) and centrifuged at 100,000× *g*. After 24 h, the separated very low-density lipoprotein (VLDL) fraction (d < 1.019 g/mL) was removed, and the density of the remaining serum was adjusted to 1.019 < d < 1.063, with subsequent centrifugation at 100,000× *g*. Post 24 h, the LDL fraction (from the density zone 1.019 < d < 1.063) was recovered and stored in a separate tube. The residual serum density was adjusted to 1.063 < d < 1.125 for the isolation of HDL_2_ using ultracentrifugation (at 100,000× *g* for 24 h). The HDL_2_ fractions were recovered and kept in separate tubes, and residual serum density was adjusted to 1.125 < d < 1.225, followed by 24 h centrifugation at 100,000× *g* to isolate the HDL_3_. The density was adjusted with NaCl and NaBr using a previously described standard method [45]. The isolated lipoprotein fractions (LDL, HDL_2_, and HDL_3_) were individually dialyzed against Tris-buffered saline [TBS, 10 mM Tris-HCl, 140 mM NaCl, and 5 mM ethylene-diamine-tetraacetic acid (EDTA), pH 8.0]. All the samples were dialyzed for 24 h. The dialyzed LDL, HDL_2_, and HDL_3_ were recovered and stored in a refrigerator for further use.

The total TC and TG in the individual lipoprotein fractions (i.e., LDL, HDL_2_, and HDL_3_) were quantified using commercially available kits (cholesterol, T-CHO, and TG, Cleantech TS-S; Wako Pure Chemical, Osaka, Japan) following the manufacturer’s instructions. The protein concentrations of the lipoproteins were determined using the Lowry protein assay, as modified by Markwell et al. [46], with bovine serum albumin (BSA) as a standard.

### 4.5. Quantification of LDL Oxidation and Agarose Gel Electrophoresis

Oxidation in the isolated LDL was quantified by measuring the concentration of oxidized species using the thiobarbituric acid reactive substance (TBARS) assay [47]. In brief, 50 μL of the LDL fraction (1 mg/mL) was mixed with 50 μL of trichloroacetic acid (20%) and 100 μL of thiobarbituric acid (0.67%) and incubated at 95 °C for 10 min. Absorbance at 560 nm was recorded, and the results are expressed as formed malondialdehyde (μM).

In addition, oxidation of LDL was verified by agarose gel electrophoresis in the native state and the induced oxidative state (in vitro oxidation) [48]. For the in vitro oxidation, LDL (1 mg/mL, 50 μL) was mixed with CuSO_4_ (10 μM, 50 μL). The mixture was incubated at 37 °C for 30 min, and then subjected to agarose gel electrophoresis. A 0.5% agarose gel was prepared, and the samples (10 μL) from the native and in vitro-oxidized LDL were loaded in the individual wells. Electrophoresis was carried out using Tris–acetate–EDTA buffer (pH 8.0) at a constant voltage of 50 V [49]. After a sufficient run (~1 h), the gel was stained with 1.25% Coomassie Brilliant Blue (CBB) solution. The separated band intensities were quantified using ImageJ software (https://image.net/ij; accessed on 6 June 2025, version 1.53).

### 4.6. Paraoxonase Assay

The paraoxonase-1 (PON-1) activity in HDL_2_ and HDL_3_ was determined by measuring the hydrolysis of paraoxon to *p*-nitrophenol and diethylphosphate, using a previously described method [50]. In brief, 20 μL of HDL_2_ or HDL_3_ (1 mg/mL equivalent protein) was mixed with 180 μL Tris-HCl (90 mM) containing paraoxon-ethyl (0.6 M). Following 60 min incubation at 37 °C, absorbance at 415 nm was recorded. The PON-1 activity was then determined by measuring the initial velocity of *p*-nitrophenol production using the molar extinction coefficient (1.7 × 10^4^/M/cm), and the PON activity is expressed as μU/L/min.

### 4.7. Ferric Ion Reducing Ability Assay

The ferric ion reducing ability (FRA) was determined using the method reported by Benzie and Strain [51]. Briefly, the FRA reagents were freshly prepared by mixing 20 mL of 0.2 M acetate buffer (pH 3.6), 2.5 mL of 10 mM 2,4,6-tripyridyl-S-triazine, and 2.5 mL of 20 mM FeCl_3_·6H_2_O. The 20 μL of HDL_2_ or HDL_3_ (1 mg/mL equivalent protein) was mixed with 180 μL of freshly prepared FRA reagent. After 60 min of incubation at room temperature, the absorbance at 593 nm was recorded. The ferrous sulfate standard curve was prepared to quantify the results as FRA (μM, ferric equivalent).

### 4.8. Electrophoresis of HDL

Electrophoresis of HDL_2_ in the denatured state was carried out to compare apoA-I band intensities using SDS-PAGE. A 15% SDS-PAGE was prepared and 10 μL of HDL_2_ samples (1 mg/mL of protein) was loaded in the well. Electrophoresis was carried out at 50 V using the Tris–acetate–EDTA buffer (pH 8.0). After a sufficient run (~1 h), the gel was stained with 0.125% CBB, after which the relative band intensity of apoA-I was compared by band scanning using Gel Doc^®^ XR (Bio-Rad, Hercules, CA, USA) and Quantity One software (version 4.5.2).

### 4.9. Glycation Extent of LDL, HDL_2_, and HDL_3_

The glycation extent of LDL, HDL_2_, and HDL_3_ was determined by reading the fluorometric intensity at 370 nm (excitation) and 440 nm (emission) employing a previously described method [52]. Glycation was measured in terms of the formed advanced glycation product (AGE) which gives the characteristic emission at 440 nm, while the exited one is at 370 nm. In brief, LDL, HDL_2_, and HDL_3_ were individually suspended with 500 μL of 0.2 M potassium phosphate–3 mM sodium azide buffer (pH 7.4) at a final concentration of 0.1 mg/mL (protein). Finally, the fluorescent intensity (FI) was examined at 370 nm (excitation) and 440 nm (emission) using the LS55 spectrofluorometer (PerkinElmer, Shelton, CT, USA), and results are depicted as glycation extent (AU, arbitrary unit).

### 4.10. Electron Microscopy

Transmission electron microscopy (TEM; Hitachi H-7800, Ibaraki, Japan) was performed at Raydel HDL Research Institute (Daegu, Republic of Korea) following a previously described method [44,53]. In brief, 5 μL of LDL/HDL_2_ samples (0.3 mg/mL in TBS) was mixed with 5 μL of 2% sodium phosphotungstate (PTA; pH 7.4) for the negative staining. Subsequently, 5 μL of the LDL/HDL_2_ suspension was placed on a formvar-coated 300-mesh copper grid and air-dried for 2 min. The excess suspension was blotted, and the grid was incubated at 50 °C for 2 h. Finally, the sample was visualized under TEM (HT-7800, Hitachi, Tokyo, Japan) at 100 kV accelerating voltage, and the images were captured at 150,000× magnification. For each sample, five independent replicates in five distinct formvar copper grids were prepared for TEM analysis, and images were captured.

Post TEM analysis, the captured images were analyzed using the Image J software (https://image.net/ij, accessed on 6 June 2025, version 1.53) for the determination of LDL/HDL_2_ particle size. For the quantification of particle size, 10 fields per image across the five independent replicates (i.e., 50 particles/sample) were analyzed, and results are depicted as mean value ± SD.

### 4.11. Data Analysis

All analyses in Table 1 were normalized by a homogeneity test of variances through Levene’s statistics. If not normalized, nonparametric statistics were used for the Kruskal–Wallis test. All values are expressed as the mean ± SD (standard deviation) for the continuous variables for the middle-aged group. All tests were two-tailed, and statistical significance was defined as *p* < 0.05.

Pearson’s correlation analysis was performed to determine the presence of a positive or negative association between the selected parameters. Statistical analyses were carried out using the SPSS statistical package version 28.0 (SPSS Inc., Chicago, IL, USA), incorporating sampling weights and adjusting for the complex survey design.

## 5. Conclusions

This cross-sectional study of 50 Korean participants revealed heightened serum TG and glucose levels associated with hypertension. An enhanced particle size of HDL (subpopulations, HDL_2_ and HDL_3_), with enhanced PON and FRA activities, shows an inverse correlation with LDL glycation, oxidative damage, and the risk of hypertension in Korean participants. Nevertheless, a comprehensive study with a large participant pool is needed to verify the current outcome and reach a confirmatory conclusion.

## Figures and Tables

**Table 1:** Anthropometric data, serum lipids, and lipoprotein profile of participants.

		Total (n = 50)	Male (n = 25)	Female (n = 25)	*p*
Age		47.0 ± 11.7	49.2 ± 11.7	44.8 ± 11.5	0.195
SBP (mmHg)		126.3 ± 15.8	132.7 ± 14.6	119.9 ± 14.4	0.003
DBP (mmHg)		74.8 ± 12.1	79.8 ± 10.8	69.8 ± 11.5	0.003
BMI		24.6 ± 4.5	26.7 ± 4.6	22.6 ± 3.3	0.003
Body fat (%)		26.3 ± 4.8	24.7 ± 4.1	27.9 ± 5.1	0.019
Subcutaneous fat (kg)		15.5 ± 4.4	16.0 ± 4.2	15.0 ± 4.7	0.411
Visceral fat (kg)		2.4 ± 1.1	2.9 ± 1.0	1.8 ± 1.0	0.001
Serum	TC (mg/dL)	208.1 ± 25.1	209.5 ± 20.9	206.7 ± 29.1	0.844
	TG (mg/dL)	92.4 ± 31.0	107.2 ± 26.8	77.7 ± 28.9	0.001
	HDL-C (mg/dL)	51.3 ± 11.8	48.6 ± 12.2	53.9 ± 11.0	0.123
	LDL-C (mg/dL)	137.3 ± 25.1	139.5 ± 23.0	135.1 ± 27.3	0.669
	TG/HDL-C (ratio)	2.0 ± 1.1	2.4 ± 1.1	1.6 ± 0.9	0.004
	LDL/HDL-C (ratio)	2.9 ± 1.1	3.2 ± 1.3	2.7 ± 0.9	0.181
	HDL-C/TC (%)	25.1 ± 6.5	23.7 ± 6.7	26.5 ± 6.1	0.138
	Glucose (mg/dL)	113.3 ± 18.9	122.0 ± 20.1	104.6 ± 4.1	0.001
	Glucose/HDL-C (ratio)	2.4 ± 0.9	2.7 ± 1.0	2.0 ± 0.6	0.009
LDL	TC (mg/dL)	126.9 ± 38.4	123.2 ± 45.0	130.2 ± 32.2	0.540
	TG (mg/dL)	24.3 ± 16.5	32.1 ± 19.6	17.4 ± 8.9	0.003
	Oxidized extent (µM)	7.2 ± 1.7	7.5 ± 1.7	7.0 ± 1.6	0.261
	Size (nm^2^)	506.5 ± 88.1	478.4 ± 89.9	534.6 ± 89.3	0.022
	Diameter (nm)	25.2 ± 2.3	24.4 ± 2.1	25.9 ± 2.2	0.022
	Glycation extent	3702 ± 502	3737 ± 591	3667 ± 404	0.628
HDL_2_	TC (mg/dL)	51.3 ± 17.0	47.2 ± 17.8	54.8 ± 15.8	0.127
	TG (mg/dL)	10.5 ± 6.9	13.7 ± 7.7	7.6 ± 4.7	0.002
	PON (µU/L/min)	6.3 ± 2.5	6.0 ± 2.8	6.5 ± 2.3	0.460
	FRA (μM)	46.4 ± 9.8	45.6 ± 11.1	47.2 ± 8.5	0.587
	Size (nm^2^)	137.6 ± 13.7	132.2 ± 15.5	143.0 ± 9.1	0.004
	Diameter (nm)	13.2 ± 0.6	12.9 ± 0.7	13.4 ± 0.4	0.002
	Glycation extent	2367 ± 435	2516 ± 486	2218 ± 323	0.014
HDL_3_	TC (mg/dL)	21.0 ± 5.5	21.2 ± 7.0	20.8 ± 3.8	0.801
	TG (mg/dL)	4.1 ± 3.4	5.1 ± 4.1	3.3 ± 2.6	0.075
	PON (µU/L/min)	31.9 ± 17.1	35.4 ± 17.6	28.5 ± 16.3	0.156
	FRA (μM)	36.8 ± 22.6	39.6 ± 20.5	33.9 ± 24.7	0.377
	Glycation extent	1527 ± 432	1677 ± 519	1377 ± 255	0.013

SBP, systolic blood pressure; DBP, diastolic blood pressure; BMI, body mass index; TC, total cholesterol; TG, triglyceride; HDL, high-density lipoprotein; HDL-C, high-density lipoprotein cholesterol; LDL-C, low-density lipoprotein cholesterol; PON, paraoxonase; FRA, ferric ion reduction ability. The *p*-value represents the statistical difference between male and female participants.

**Table 2:** Pearson correlation analysis of anthropometric, serum lipid, and lipoprotein parameters with SBP and DBP.

		SBP (mmHg)		DBP (mmHg)	
		*r*	*p*	*r*	*p*
Body	Age	0.245	0.087	0.132	0.360
	BMI	0.362	0.011	0.360	0.011
	Body fat (%)	0.135	0.354	0.125	0.393
	Subcutaneous fat (kg)	0.248	0.386	0.235	0.104
	Visceral fat (kg)	0.407	0.004	0.381	0.007
Serum	TC (mg/dL)	0.222	0.121	0.117	0.419
	TG (mg/dL)	0.575	<0.001	0.553	<0.001
	HDL-C (mg/dL)	−0.238	0.096	−0.234	0.102
	LDL-C (mg/dL)	0.226	0.115	0.139	0.336
	TG/HDL-C (ratio)	0.476	<0.001	0.418	0.002
	LDL/HDL-C (ratio)	0.242	0.091	0.157	0.275
	HDL-C/TC (%)	−0.298	0.036	−0.243	0.089
	Glucose (mg/dL)	0.525	<0.001	0.504	<0.001
	Glucose/HDL-C (ratio)	0.399	0.004	0.352	0.012
LDL	TC (mg/dL)	0.063	0.672	0.056	0.710
	TG (mg/dL)	0.506	<0.001	0.448	0.002
	Oxidized extent (MDA)	0.617	<0.001	0.672	<0.001
	Size (nm^2^)	−0.436	0.002	−0.501	<0.001
	Diameter (nm)	−0.447	<0.001	−0.501	<0.001
	Glycation extent	0.464	<0.001	0.542	<0.001
HDL_2_	TC (mg/dL)	−0.069	0.644	−0.066	0.660
	TG (mg/dL)	0.474	<0.001	0.444	0.002
	PON (µU/L/min)	−0.073	0.613	−0.047	0.746
	FRA (µM)	−0.050	0.733	−0.044	0.762
	Size (nm^2^)	−0.486	<0.001	−0.443	0.001
	Diameter (nm)	−0.500	<0.001	−0.422	0.002
	Glycation extent	0.557	<0.001	0.557	<0.001
HDL_3_	TC (mg/dL)	0.035	<0.001	−0.007	0.961
	TG (mg/dL)	0.252	0.088	0.258	0.079
	PON (µU/L/min)	0.078	0.590	0.061	0.675
	FRA (µM)	0.088	0.545	0.048	0.740
	Glycation extent	0.402	0.004	0.428	0.002

SBP, systolic blood pressure; DBP, diastolic blood pressure; BMI, body mass index; TC, total cholesterol; TG, triglyceride; HDL, high-density lipoprotein; HDL-C, high-density lipoprotein cholesterol; LDL-C, low-density lipoprotein cholesterol; PON, paraoxonase; FRA, ferric ion reduction ability. The *p*-value represents the statistical significance of Pearson correlation analysis (two-tailed).

## Data Availability

The original contributions presented in this study are included in the article/Supplementary Materials. Further inquiries can be directed to the corresponding author.

## Supplementary Materials

The following supporting information can be downloaded at https://www.mdpi.com/article/10.3390/ijms27021108/s1.
