# Supplementary material for: Enhanced Qualities of High-Density Lipoproteins (HDLs) with Antioxidant Abilities Are Associated with Lower Susceptibility of Hypertension in Middle-Aged Korean Participants: Impaired HDL Quality and Hypertension Risk
Source: Int J Mol Sci. 2026 Jan 22;27(2):1108. doi: 10.3390/ijms27021108 (PMC12842381; doi:10.3390/ijms27021108)
Supplement: Supplementary file 1 [file ijms-27-01108-s001.zip › ijms-4070686-supplementary.pdf]

Supplementary Figures

Supplementary Figure S1

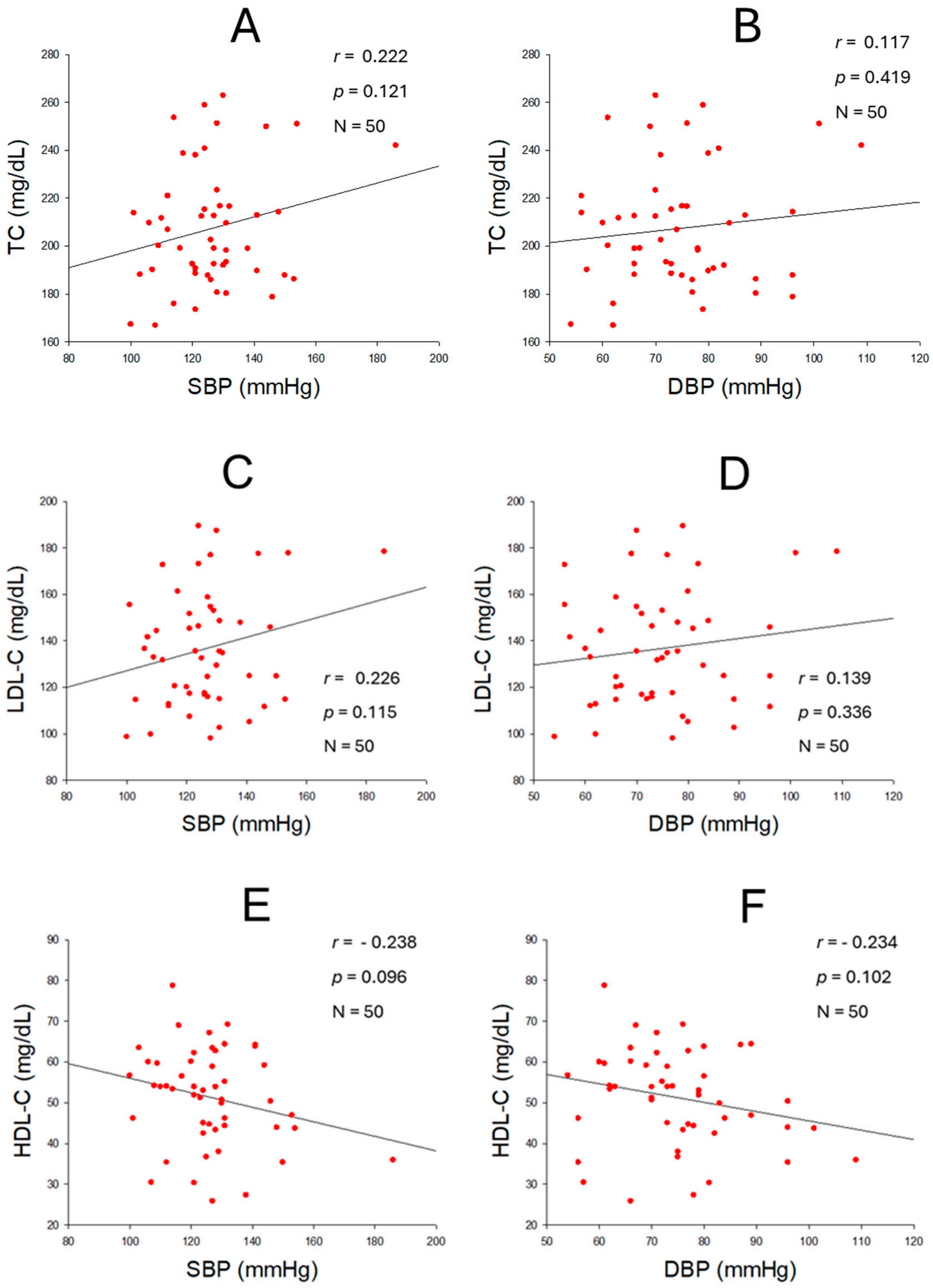

Supplementary Figure S1. Correlation analysis of serum lipid profile and blood pressure (BP).

(A) & (B) correlation between serum total cholesterol (TC) with systolic BP (SBP) and diastolic BP (DBP), respectively. (C) & (D) correlation between serum high-density lipoprotein cholesterol (HDL-C) with systolic BP (SBP) and diastolic BP (DBP), respectively. (E) & (F) correlation between serum low-density lipoprotein cholesterol (LDL-C) with systolic BP (SBP) and diastolic BP (DBP), respectively.

Supplementary Figure S2

(A) M1 (34 year-old)

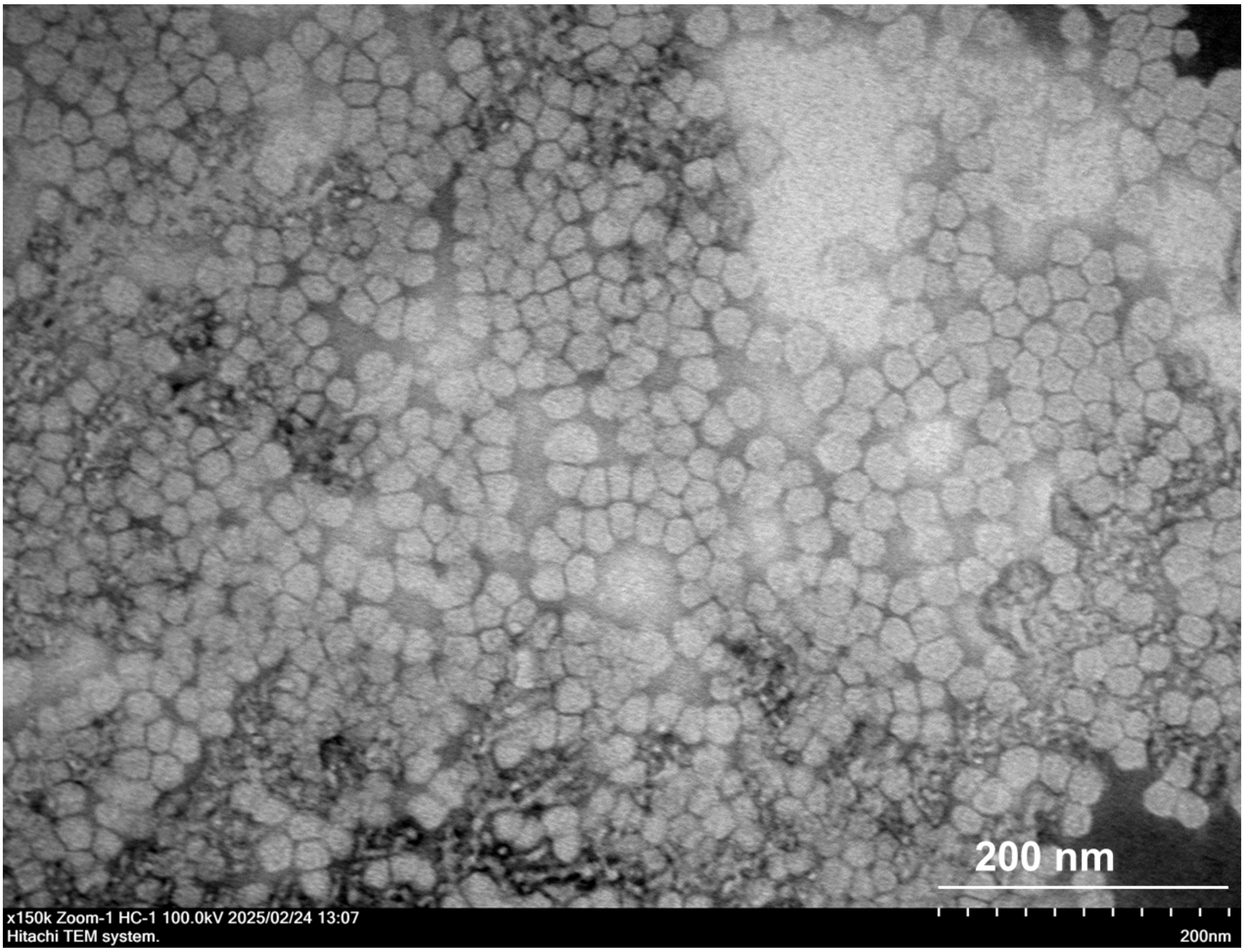

Diameter: 25.8 ± 2.5 nm; Size: 591.1 ± 88.9 nm^2

(B) M2 (47 year-old)

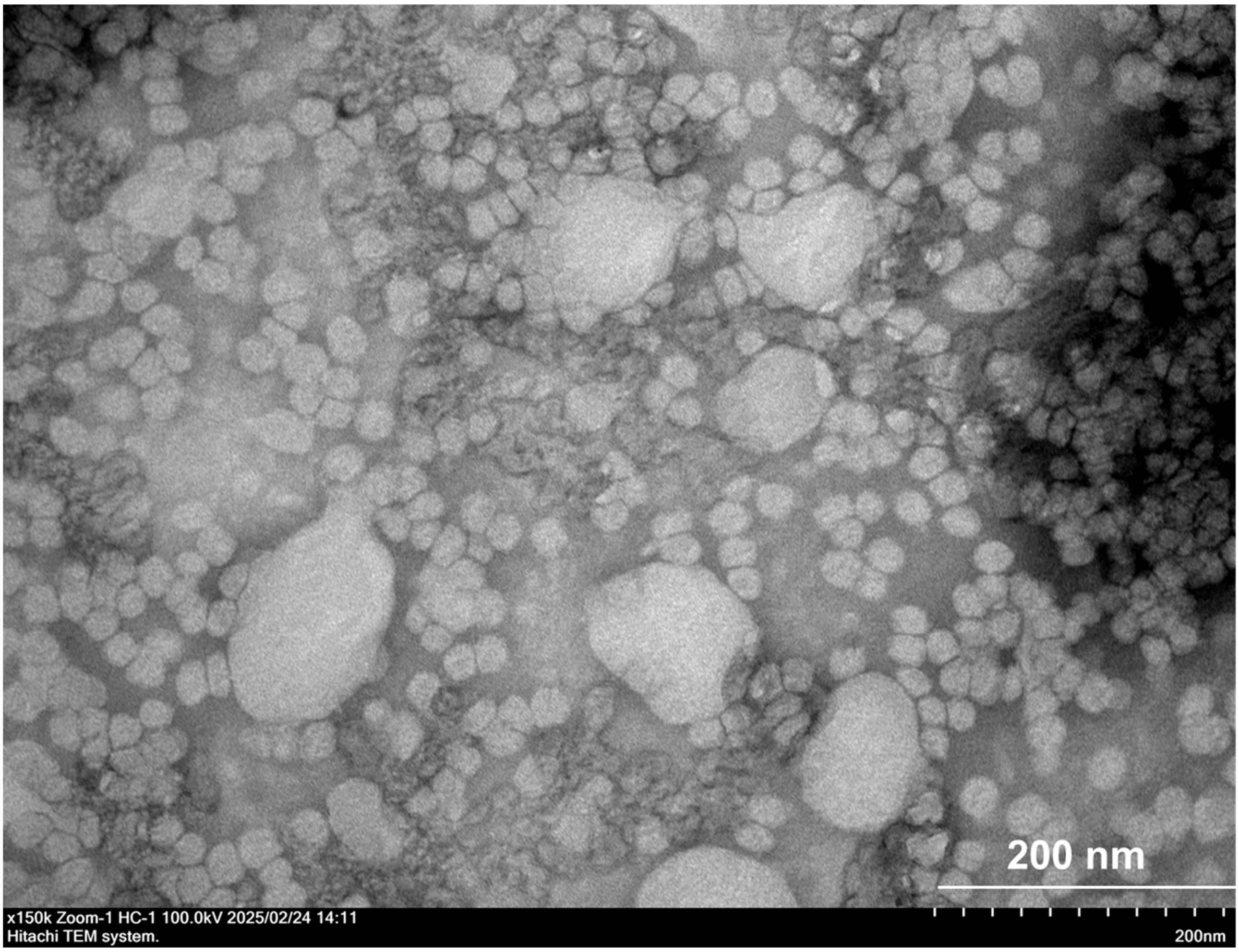

Diameter: 24.2 ± 3.4 nm; Size: 471.5 ± 120.7 nm^2

(C) M1 (51 year-old)

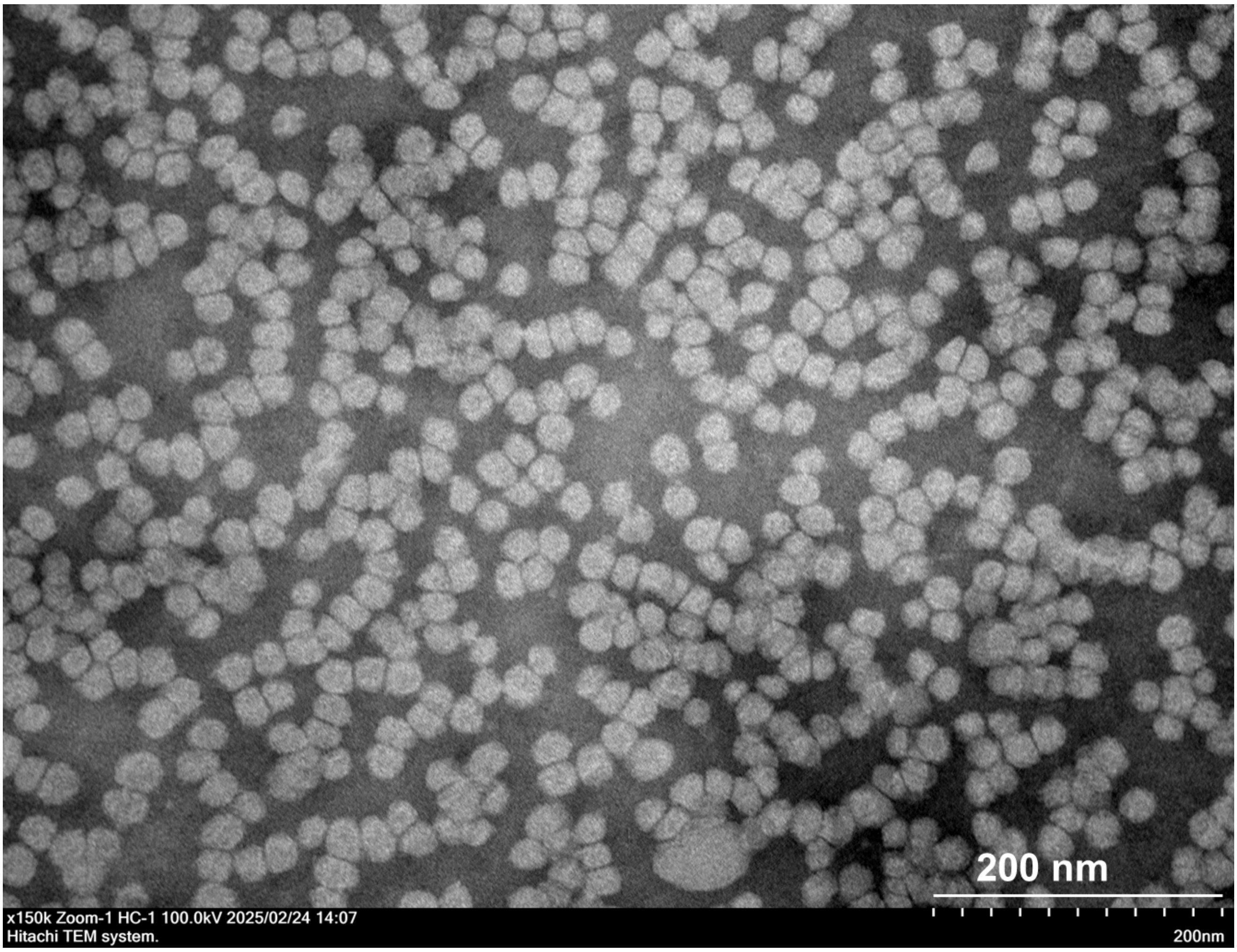

Diameter: 25.6 ± 2.2 nm; Size: 571.8 ± 94.1 nm^2

(D) M4 (51year-old)

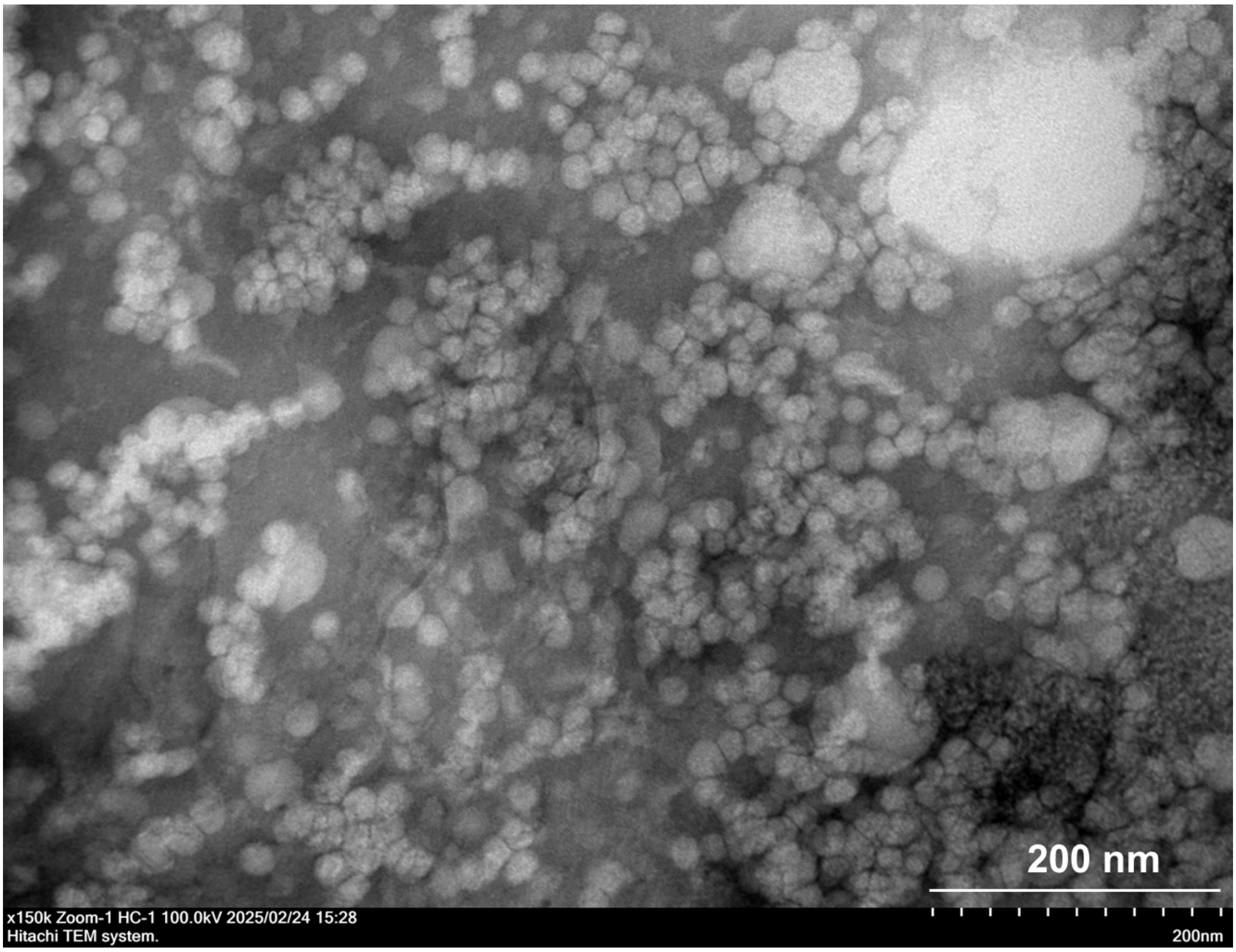

Diameter: 24.4 ± 3.6 nm; Size: 459.7 ± 137.6 nm^2

Supplementary Figure S2. Transmission electron microscopic (TEM) images of low-density lipoprotein (LDL) particles of male participants (A) and (B) normotensive participants (M1,M2) and (C) and (D) hypertensive participants (M2 and M4).

Supplementary Figure S3

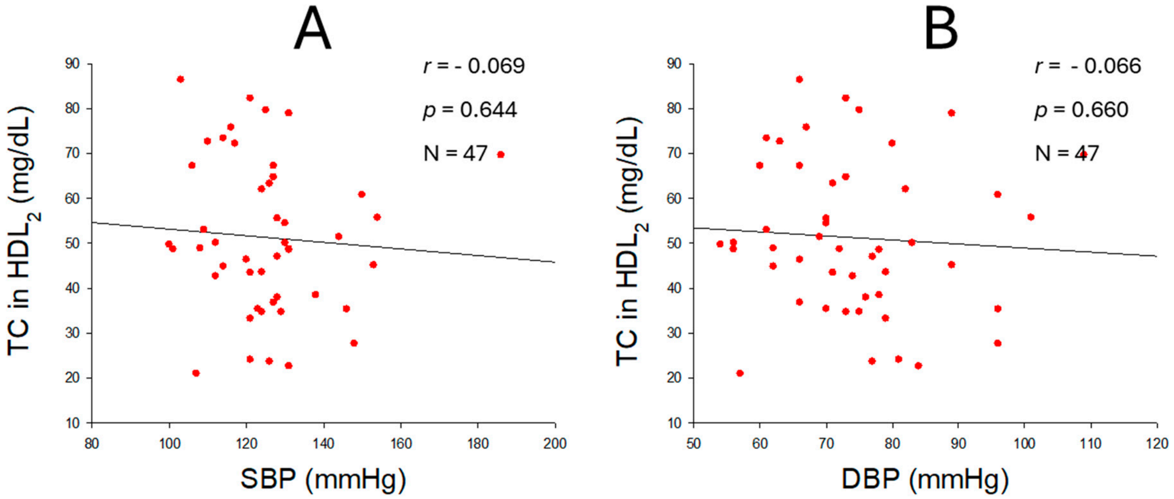

Supplementary Figure S3. Correlation analysis of total cholesterol (TC) in high-density lipoprotein (HDL_2) with (A) systolic blood pressure (SBP) and diastolic blood pressure (DBP), respectively.

Supplementary Figure S4

(A) M1 (34 year-old)

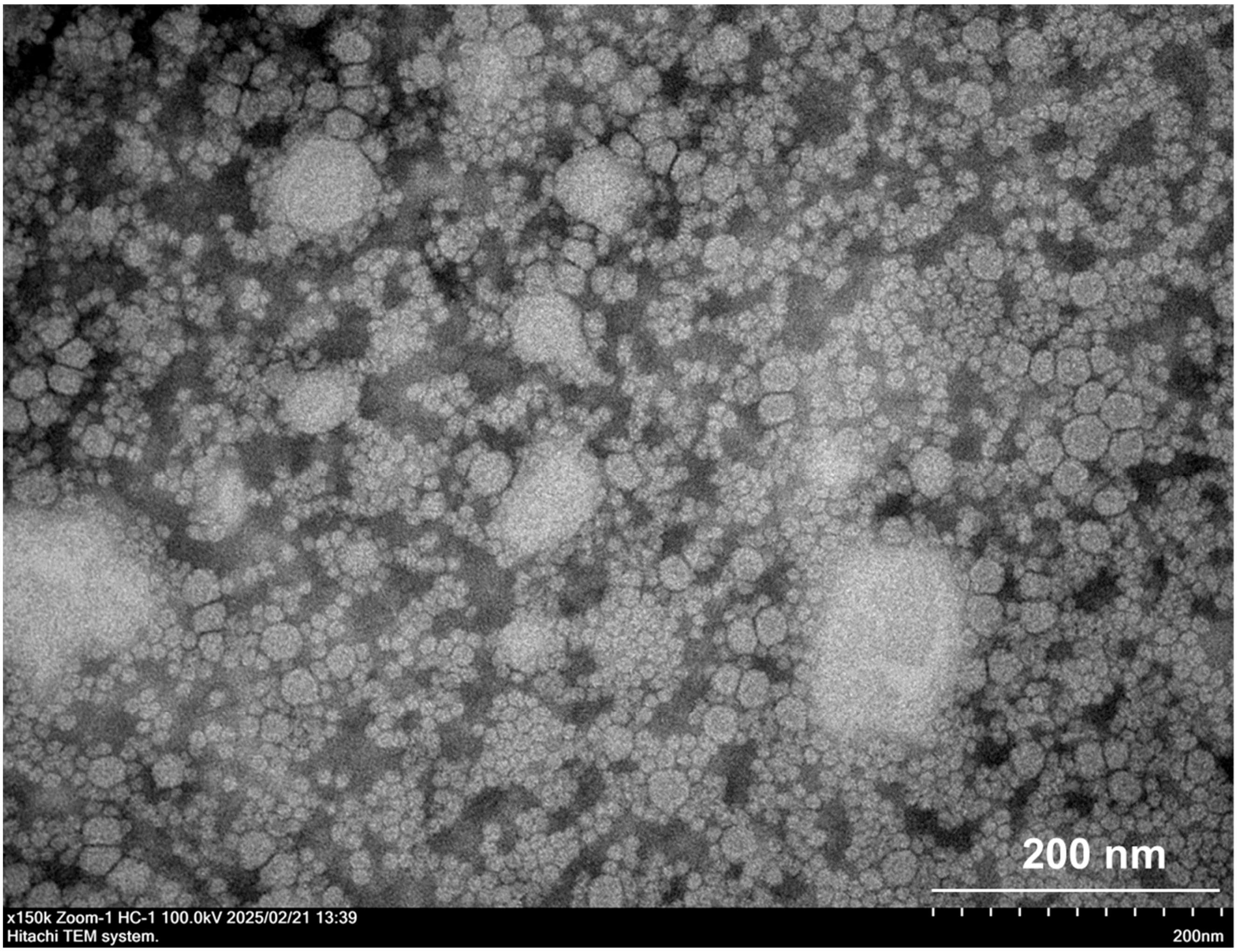

Diameter: 13.0 ± 3.7 nm; Size: 123.9 ± 12.8 nm^2

(B) M2 (47 year-old)

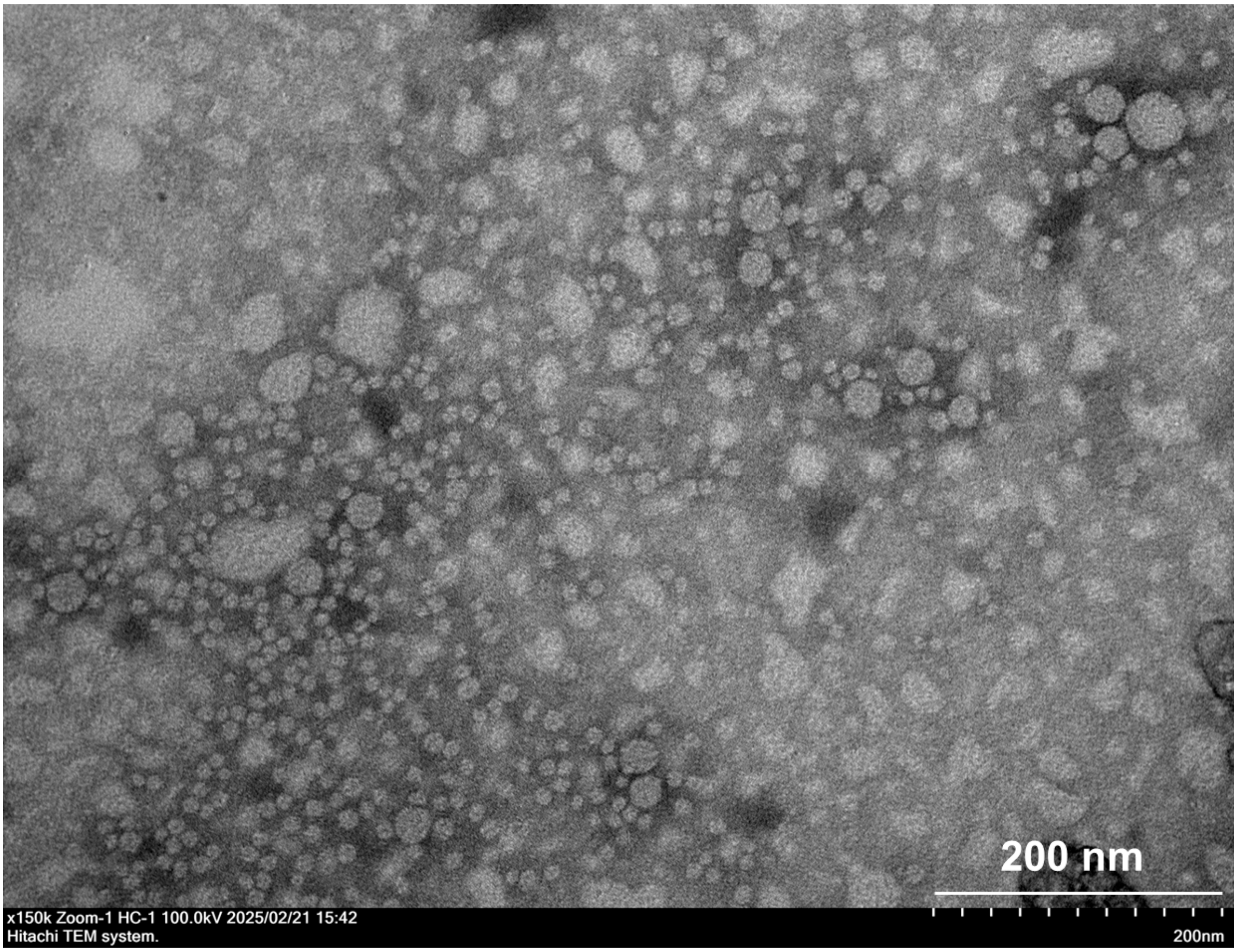

Diameter: 11.9 ± 0.5 nm; Size: 117.7 ± 10.1 nm^2

(C) M3 (51 year-old)

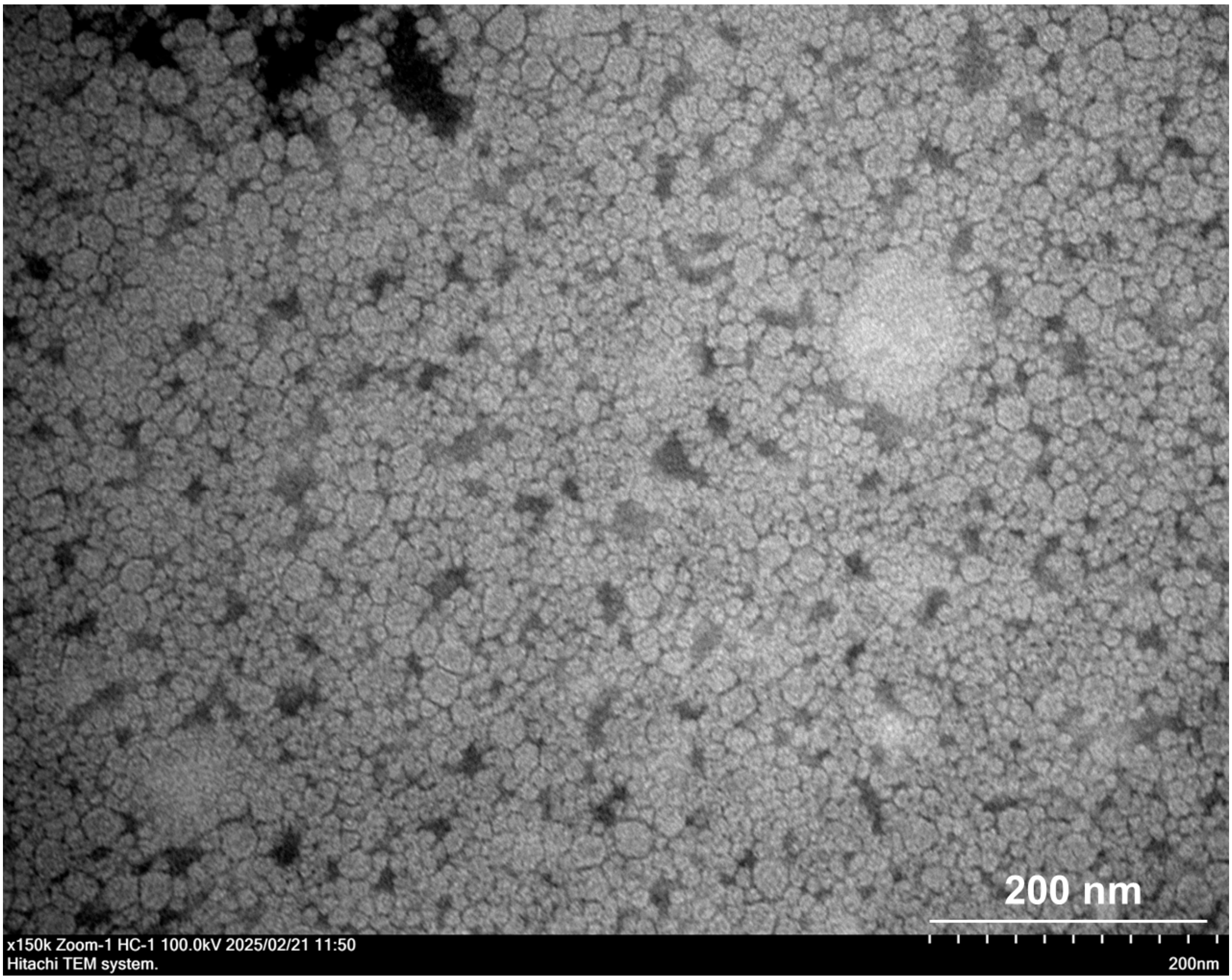

Diameter: 13.2 ± 0.9 nm; Size: 125.9 ± 17.7 nm^2

(D) M4 (51 year-old)

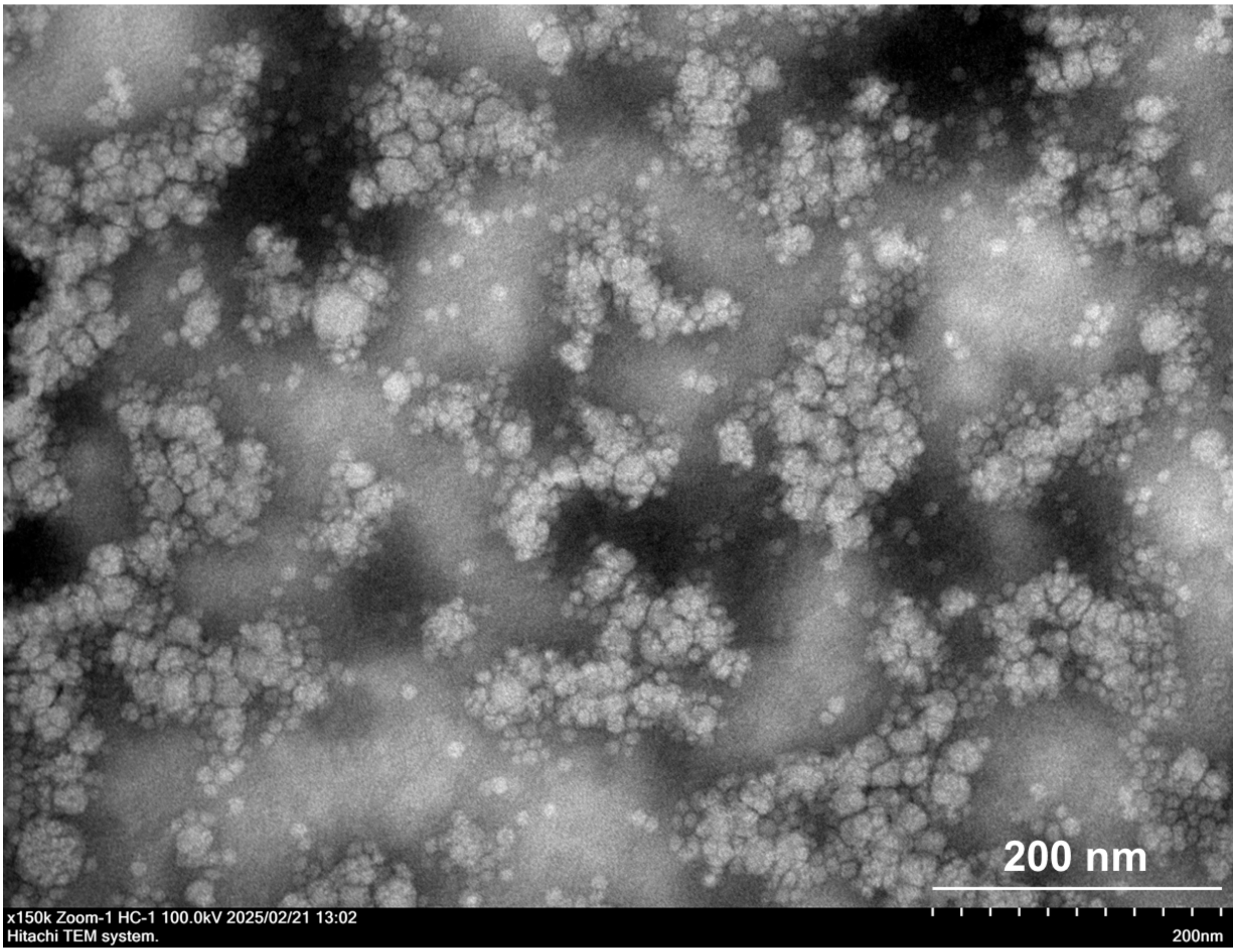

Diameter: 12.2 ± 0.7 nm; Size: 118.4 ± 14.0 nm^2

Supplementary Figure S4. Transmission electron microscopic (TEM) images of high-density lipoprotein (HDL_2) particles of male participants (A) and (B) normotensive participants (M1, M2) and (C) and (D) hypertensive participants (M2 and M4).

Supplementary Figure S5

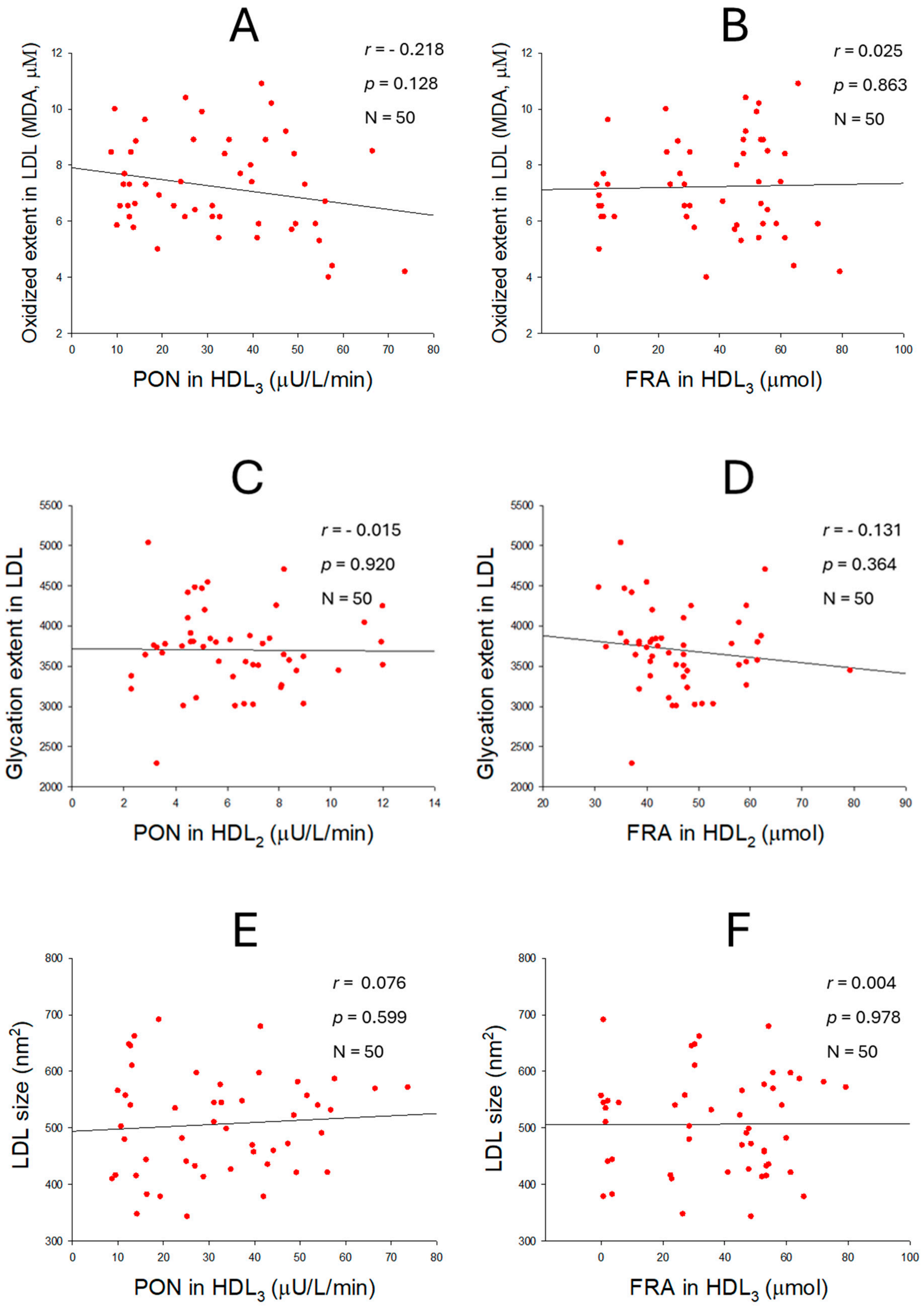

Supplementary Figure S5. Correlation analysis of low-density lipoprotein (LDL) qualities and antioxidant abilities in high-density lipoprotein (HDL_2) and high-density lipoprotein (HDL_3). (A) & (B) correlation between oxidized extent in LDL with HDL_3 associated paraoxonase (PON) and ferric ion reduction ability (FRA), respectively. (C) & (D) Correlation between glycation extent in LDL with HDL_2 associated paraoxonase (PON) and ferric ion reduction ability (FRA), respectively. (E) & (F) Correlation between size of LDL with HDL_3 paraoxonase (PON) and ferric ion reduction ability (FRA), respectively.
